# Poor chemical and microbiological quality of the commercial milk thistle-based dietary supplements may account for their reported unsatisfactory and non-reproducible clinical outcomes

**DOI:** 10.1038/s41598-019-47250-0

**Published:** 2019-07-31

**Authors:** Marie Fenclova, Alena Novakova, Jitka Viktorova, Petra Jonatova, Zbynek Dzuman, Tomas Ruml, Vladimir Kren, Jana Hajslova, Libor Vitek, Milena Stranska-Zachariasova

**Affiliations:** 1 Department of Food Analysis and Nutrition, University of Chemistry and Technology, Technicka 3, 16628 Prague 6, Czech Republic; 2 Department of Biochemistry and Microbiology, University of Chemistry and Technology, Technicka 3, 16628 Prague 6, Czech Republic; 3 Laboratory of Biotransformation, Institute of Microbiology of the Czech Academy of Sciences, Vídeňská 1082, 14000 Prague 6, Czech Republic; 4 Institute of Medical Biochemistry and Laboratory Diagnostics and 4th Department of Internal Medicine, 1st Faculty of Medicine, Charles University, Katerinska 32, 12108 Prague 2, Czech Republic

**Keywords:** Biochemistry, Risk factors

## Abstract

Herbal-based dietary supplements have become increasingly popular. The extract from milk thistle (*Silybum marianum)*, is often used for the treatment of liver diseases. However, serious concerns exist regarding the efficacy, composition, as well as the safety of these over-the-counter preparations. Therefore, the aim of the present study was to investigate the composition as well as chemical and biological safety of 26 milk thistle-based dietary supplements purchased from both the U.S. and Czech markets between 2016 and 2017. The study was focused on a determination of the composition of active ingredients, as well as analyses of possible contaminants including: mycotoxins, plant alkaloids, and pesticide residues, as well as the microbial purity. High-throughput analyses were performed using advanced U-HPLC-HRMS techniques. Large differences in the silymarin content were observed among individual milk thistle preparations, often in contrast with the information provided by the manufacturers. In addition, substantial inter-batch differences in silymarin content were also demonstrated. In all milk thistle preparations tested, large numbers and high concentrations of mycotoxins and several pesticides, as well as the substantial presence of microbiological contamination were detected, pointing to serious safety issues. In conclusion, our results strongly indicate the need for strict controls of the composition, chemical contaminants, as well as the microbiological purity of commercial milk thistle extracts used for the treatment of liver diseases. Poor definition of these preparations together with contamination by biologically active substances may not only account for the inconsistency of clinical observations, but also be responsible for possible herbal-based dietary supplements-induced liver injury.

## Introduction

It has been reported that approximately 20% to 50% of European and US adults use herbal-based dietary supplements^1^, further that this trend is steadily increasing, with billions of dollars spent annually on herbal-based dietary supplements^2^. The extract of milk thistle, *Silybum marianum*, is among the most common and one of the six best-selling herbal-based dietary supplements in the US^3^. Botanically, milk thistle is a tall purple flowering plant belonging to the *Aster* family and in herbal medicine is often used for the treatment of liver diseases. The major component represented in milk thistle-based dietary supplements is silymarin, which is a complex of flavonolignans silybin A and B, isosilybin A and B, silydianin, silychristin, isosilychristin, together with the flavonoid taxifolin. The aforementioned compounds are found together with an additional ~30% component, which contains a somewhat undefined yet potentially bioactive polymeric (polyphenolic) fraction^4,5^. The continuing and increasing popularity of silymarin for chemoprevention or the treatment of liver diseases has led to several systematic reviews of the efficacy of these silymarin preparations^6–8^. Although most of experimental reports as well as some clinical data suggest it does play a beneficial role^6,7,9^, silymarin is generally considered to have negligible clinical importance^2,8,10,11^. The main limitation seems to be the lack of properly controlled clinical trials, with standardized therapeutic efficacy assessment methods used; along with the poor definition of silymarin preparations used in such clinical studies, which may differ substantially one from another – and possibly also from what is declared by the manufacturers^12,13^.

Nevertheless, a large proportion of liver disease (27 to 43%) or cancer patients (26 to 87%) use this hepatoprotective herbal-based dietary supplements hoping to improve their health and quality of life^14–17^. However, although these preparations have been popularized as effective cancer preventive agents, considering the lack of clear evidence of efficacy as well as food safety^18^, a more cautious approach by consumers would be appropriate. For example, the study of Lee *et al*. focused on measuring the prevalence of dietary supplement use by patients suffering from hepatocellular carcinoma revealed that patients who used milk thistle had marginally poorer survival than non-users did^14^.

In this context, the important fact is that dietary supplements are classified as foods^19,20^, thereby considerably less strict rules are being applied before the products enter the market compared to drugs, particularly in terms of accurate composition parameters as well as safety issues.

Dietary supplements are placed onto the marketplace with limited or sometimes no research on how effectively they work. In the U.S., the Food and Drug Administration (FDA) has not approved milk thistle as a treatment agent for cancer nor any other diseases, and it is only available as a dietary supplement. In Europe, the situation is quite similar.

The other potential problem is the safety issue. Several studies have referred to microbial contamination of milk thistle-based supplements by toxinogenic fungi^21^, and the presence of mycotoxins in various herbal-based dietary supplements have recently been published^22–24^. It is noteworthy that in addition to mycotoxins, it is also not exceptional to find the presence of pesticide residues^25,26^ or toxic plant alkaloids^27^ in the herbal extracts. And last but not least is the (to date often unexplored) effects of the biologically active substituents of herbal supplements on various metabolic pathways, or their interactions with standard treatment drugs^2^. The resulting adverse effects, or the overall combined toxicity of mixtures of undesirable chemicals present may significantly influence the proposed effects of beneficial health compounds in the organism. As concluded in a recent review paper by Navarro *et al*., there is a substantive need for “improvements in regulatory oversight of non-prescription products to guarantee their constituents and ensure purity and safety^28^”.

Therefore, the aim of the present study was to investigate the composition of 26 milk thistle-based dietary supplements purchased on the US and Czech markets between 2016 and 2017 to ascertain whether the declared information matched reality, as well as to control the chemical and microbiological safety factors.

## Materials and Methods

### Analytical standards and chemicals

The analytical standards of 323 pesticides, 55 mycotoxins, and 11 plant alkaloids (for a complete list see Suppl. Table S1) were purchased from Sigma-Aldrich (Prague, Czech Republic), Merck (Prague, Czech Republic), PhytoLab (Vestenbergsgreuth, Germany), GeneTiCa (Prague, Czech Republic), Dynex Technologies (Prague, Czech Republic), and Chromservis (Prague, Czech Republic), respectively. The analytical standards of silybin A; silybin B; isosilybin A; isosilybin B; 2,3-dehydrosilybin; silychristin; silydianin; and taxifolin (Suppl. Fig. S1) were isolated from commercially available silymarin (purchased from Liaoning Senrong Pharmaceutical, Panjin, People’s Republic of China, batch no. 120501), according to published method^29^. The internal reference sample of milk thistle-based dietary supplement; *i.e*., dried milk thistle extract (containing 139 ± 17 mg/g of silybin A, 179 ± 23 mg/g of silybin B, 38 ± 5.2 mg/g of isosilybin A, 8.8 ± 0.9 mg/g of isosilybin B, 2.5 ± 0.3 mg/g of 2,3-dehydrosilybin, 180 ± 31 mg/g of silychristin, 72 ± 8.3 mg/g of silydianin, 13.5 ± 6.2 mg/g of taxifolin, 136 ± 8 µg/kg of T2 toxin, 65 ± 4.2 µg/kg of zearalenone (ZEA), 212 ± 11 µg/kg of enniatine B, 103 ± 9.5 µg/kg of enniatine B1, 32 ± 4.9 µg/kg of enniatine A, 85 ± 7.1 µg/kg of enniatine A1, 83 ± 5.5 µg/kg of beauvericine, 209 ± 18 µg/kg of alternariol (AOH), 361 ± 19 µg/kg of alternariol methylether (AME), and 87 ± 5.9 µg/kg of tentoxin (TEN); without contamination by pesticides and tropane alkaloids) was available from our previous study^30^; the reference values were calculated as mean values from repeated analyses (n = 40) by the methods described below, obtained over a long period of time. All standards and reference materials were stored at -20 °C before usage. The HPLC grade acetonitrile, ammonium acetate, ammonium formate, sodium chloride, and magnesium sulfate were obtained from Sigma-Aldrich. HPLC grade ethanol, methanol, and hexane were purchased from Merck (Darmstadt, Germany); formic acid from Penta (Chrudim, CZ); and Bondesil-C18 sorbent from Varian (Palo Alto, CA, USA).

### Samples

The samples of milk thistle-based dietary supplements were purchased from Czech and U.S. markets between 2016 and 2017 (Table 1). The preparations were primarily comprised of capsules with dried or oil-based milk thistle extracts (this means an extract from *S. marianum fruits (cypselae)*). For some brands, different sample batches were collected to assess possible inter-individual variability of the chemical composition within the same preparations.

**Table 1:** Description of the investigated milk thistle samples (as declared by producers).

Sample No.	Sample code (country of origin, brand-batch No)	Sampling year	Application form	Composition of preparation, as specified by producer (milk thistle extract)^d^	Composition of preparation, as specified by producer (other components)
1	**USA 1-I**	2016	capsules with dried powder	Milk thistle seed extract (*Silybum marianum*) 140 mg in 1 capsule - silymarin (by UV) 98 mg (i.e. 70% of silymarin)^a^	
2	**USA 1-II**	2017	capsules with dried powder	Milk thistle seed extract (*Silybum marianum*) 140 mg in 1 capsule - silymarin (by UV) 98 mg (i.e. 70% of silymarin) ^a^	
3	**USA 2**	2016	encapsulated oily paste	Milk thistle extract (*Silybum marianum*-seed) 250 mg in 1 capsule - a 4:1 extract, equivalent to 1000 mg of milk thistle seed (note: % of silymarin not possible to calculate)	
4	**USA 3-I**	2016	capsules with dried powder	Milk thistle extract (*Silybum marianum*-seed) 525 mg in 3 capsules - standardized to contain 80% silymarin, 420 mg	
5	**USA 3-II**	2017	capsules with dried powder	Milk thistle extract (*Silybum marianum*-seed) 525 mg in 3 capsules - standardized to contain 80% silymarin, 420 mg	
6	**USA 4-I**	2016	capsules with dried powder	Milk thistle extract (*Silybum marianum*-seed) 175 mg in 1 capsule - standardized to contain 80% silymarin, 140 mg	
7	**USA 4-II**	2017	capsules with dried powder	Milk thistle extract (*Silybum marianum*-seed) 175 mg in 1 capsule - standardized to contain 80% silymarin, 140 mg	
8	**USA 4-III**	2017	capsules with dried powder	Milk thistle extract (*Silybum marianum*-seed) 175 mg in 1 capsule - standardized to contain 80% silymarin, 140 mg	
9	**USA 4-IV**^**b**^	2017	encapsulated oily paste^b^	Milk thistle extract (*Silybum marianum*-seed) 250 mg in 1 capsule - a 4:1 extract, equivalent to 1000 mg whole herb (note: % of silymarin not possible to calculate)	
10	**USA 5-I**	2016	capsules with dried powder	Milk thistle seed extract 175 mg in 1 capsule - standardized to 80% silymarin (140 mg)	Blessed thistle (stem, leaf, flower) 120 mg in 1 capsule
11	**USA 5-II**	2017	capsules with dried powder	Milk thistle seed extract 175 mg in 1 capsule - standardized to 80% silymarin (140 mg)	Blessed thistle (stem, leaf, flower) 120 mg in 1 capsule
12	**USA 5-III**	2017	capsules with dried powder	Milk thistle seed extract 175 mg in 1 capsule - standardized to 80% silymarin (140 mg)	Blessed thistle (stem, leaf, flower) 120 mg in 1 capsule
13	**USA 6-I**	2016	capsules with dried powder	Milk thistle extract (*Silybum marianum*-seed) 240 mg in 2 capsules - standardized to contain 80% silymarin, 192 mg	XTRA Premium Blend® 240 mg in 2 capsules, Fennel (*Foeniculum vulgare* - seed), Dandelion (*Taraxacum officinale* - root), Licorice (*Glycyrrhiza glabra* - root)
14	**USA 6-II**	2017	capsules with dried powder	Milk thistle extract (*Silybum marianum*-seed) 240 mg in 2 capsules - standardized to contain 80% silymarin, 192 mg	XTRA Premium Blend® 240 mg in 2 capsules, Fennel (*Foeniculum vulgare* - seed), Dandelion (*Taraxacum officinale* - root), Licorice (*Glycyrrhiza glabra* - root)
15	**USA 6-III**	2017	capsules with dried powder	Milk thistle extract (*Silybum marianum*-seed) 240 mg in 2 capsules - standardized to contain 80% silymarin, 192 mg	XTRA Premium Blend® 240 mg in 2 capsules, Fennel (*Foeniculum vulgare* - seed), Dandelion (*Taraxacum officinale* - root), Licorice (*Glycyrrhiza glabra* - root)
16	**USA 6-IV**	2017	capsules with dried powder	Milk thistle extract (*Silybum marianum*-seed) 240 mg in 2 capsules - standardized to contain 80% silymarin, 192 mg	XTRA Premium Blend® 240 mg in 2 capsules, Fennel (*Foeniculum vulgare* - seed), Dandelion (*Taraxacum officinale* - root), Licorice (*Glycyrrhiza glabra* - root)
17	**USA 7-I**	2017	capsules with dried powder	Milk thistle extract (*Silybum marianum*-seed) 175 mg in 1 capsule - standardized to contain 80% silymarin, 140 mg	
18	**USA 7-II**	2017	capsules with dried powder	Milk thistle extract (*Silybum marianum*-seed) 175 mg in 1 capsule - standardized to contain 80% silymarin, 140 mg	
19	**USA 8**	2017	capsules with dried powder	Milk thistle extract (*Silybum marianum*-seed) 250 mg in 1 capsule - standardized to contain a minimum of 80% silymarin	
20	**CZ 1**	2016	capsules with dried powder	Milk thistle extract (*Silybum marianum* - seed) 250 mg in 1 capsule **-** standardized to contain 70% silymarin	*Schizandra chinensis* extract – 100 mg in 1 capsule
21	**CZ 2**	2016	capsules with dried powder	Milk thistle extract (*Silybum marianum* - seed) standardized to contain 80% of silymarin complex, 250 mg of silymarin in 1 capsule	
22	**CZ 3**	2016	capsules with dried powder	Milk thistle standardized extract (*Silybum marianum* – seed), 100 mg of silymarin in 1 capsule (note: % of silymarin not possible to calculate)	
23	**CZ 4**	2016	capsules with dried powder	Milk thistle powder (*Silybum marianum* – freeze-grinded seeds) - 390 mg in 1 capsule standardized to contain a minimum of 1.5% silymarin	
24	**CZ 5**	2016	capsules with dried powder	Milk thistle extract (*Silybum marianum* - seed), 150 mg in 1 capsule (note: % of silymarin not possible to calculate)	*Cordyceps sinensis* extract – 50 mg in 1 capsule, *Scutellaria baicalensis* extract - 50 mg in one capsule
25	**CZ 6**	2016	encapsulated oily paste	Silymarin from milk thistle standardized extract (*Silybum marianum* - seed), 100 mg of silymarin in 2 capsules^c^	
26	**CZ 7**	2016	encapsulated oily paste	Milk thistle extract (*Silybum marianum* - seed), 175 mg in 1 capsule - standardized to contain a minimum of 80% Silymarin	

^a^% of silymarin calculated from the declared amounts of *Silybum marianum* extract and declared amount of silymarin.

^b^The USA producer No 4 changed the production technology without indicating this on the product packaging.

^c^Amount of milk thistle extract is unknown.

^d^The declared milk thistle-based extract contained approx. 20-100% of the real weight of the internal content of the capsule.

### Determination of silymarin flavonoids/flavonolignans

Prior to the extraction procedure, the internal contents of capsules (n = 10) were taken out, weighed separately, and then properly mixed together in a 50 mL polytetrafluorethylene centrifuge tube (Merci, Czech Republic) to obtain homogenized sample material. For the analysis of silymarin, an optimized method developed and validated in the authors’ laboratory was used. In brief, an amount of 1 (± 0.01) g of the homogeneous material from inside of the capsules was weighed into a 50 mL polytetrafluorethylene centrifuge tube and extracted by shaking with 15 mL of ethanol for 60 min. After centrifugation (13,000 g, 5 min), the supernatant was transferred into a 50 mL volumetric flask, and a new amount of 15 mL of ethanol was added to the solid sample. This was repeated twice again, and approx. 45 mL of pooled extract was filled into the volumetric flask (50 mL) with ethanol. Then the extract was micro-filtered (0.2 µm PTFE microfilter, Alltech, USA), diluted 10-, 100- and 1000- times from the original extract, and transferred into a 2 mL autosampler vial for follow-up ultra-high performance liquid chromatographic/mass spectrometric (U-HPLC-MS) analyses. These were performed by utilizing a Dionex UltiMate 3000 ultra-high performance liquid chromatograph (Thermo Scientific, Sunnyvale, CA, USA) with a reversed phase Accucore^TM^ aQ analytical column (150 mm × 2.1 mm; i.d. 2.6 µm; Thermo Scientific) and a Q-Exactive^TM^ high resolution tandem mass spectrometer (Thermo Scientific, Bremen, Germany). The analytes present in the samples were identified based on their exact masses (m/z) of respective ions and retention times, when compared to the related analytical standards. The chromatogram of extracted ions of the silymarin components is provided in Suppl. Fig. S2.

The total quantitative isolation of silymarin (*i.e*., 100% recovery) was assured by the repeated use of the procedure described above, where during method optimization, the presence of silymarin in the successive extracts was controlled (data not shown). For purposes of quantification, a calibration batch (calibration points at levels: 1, 2.5, 5, 10, 25, 50, 100, 250, 500, 1000, and 2500 μg/L) with a mixture of: silybin A; silybin B; isosilybin A; isosilybin B; 2,3-dehydrosilybin; silychristin; silydianin; and taxifolin was prepared by placing appropriate volumes of the basic standards in ethanol into vials, and then adding additional ethanol to obtain the desired concentrations.

The repeatability of the method, expressed as a relative standard deviation (RSD), was assessed by repeated analyses (n = 7) of an internal reference sample of milk thistle-based dietary supplement; for individual analytes these were between 2.7–5.4%. The limits of quantification (LOQs) of silymarin components were estimated as the lowest concentration levels of the calibration batch providing long-term stable signals, and were 0.75, 0.75, 0.5, 0.5, 0.25, 2.5, 1.25 and 1.25 μg/g for silybin A, silybin B, isosilybin A, isosilybin B, 2,3-dehydrosilybin, silychristin, silydianin, and taxifolin, respectively.

### Determination of mycotoxins, plant alkaloids, and pesticide residues

Prior to the extraction procedure, the internal contents of capsules (n = 10) were taken out, weighed separately, and properly mixed together in a 50 mL polytetrafluorethylene centrifuge tube (Merci, Czech Rrepublic) to obtain homogenized sample material. The samples were processed as previously described^24,30^. Briefly, a representative sample (1 ± 0.01 g) was weighed into a polytetrafluorethylene centrifuge tube and mixed with 10 mL of 1% aqueous formic acid. The matrix was allowed to soak for 30 min, and then extracted 30 min by shaking with 10 mL of acetonitrile. Next, 1 g of sodium chloride and 4 g of magnesium sulfate were added and the tube was again shaken for 1 min. After centrifugation (5 min at 13,000 g), a 2 mL aliquot of the upper acetonitrile layer was taken for dispersive solid phase extraction clean-up in a smaller tube containing 100 mg of Bondesil-C18 sorbent and 300 mg of magnesium sulfate. The tube was shaken by hand for approximately 1 min and then centrifuged for 5 min (13,000 g). An aliquot of the upper acetonitrile layer was micro-filtered (0.2 µm PTFE microfilter, Alltech, USA) and transferred into a 2 mL autosampler vial for follow-up U-HPLC-MS analyses. As for any oily paste, the sample (1 g) was first shaken for 5 min with 3 mL of hexane to remove lipids. After this step, the same procedure was followed as described above.

For the U-HPLC-HRMS analyses, our previously developed method using a Dionex UltiMate 3000 UHPLC chromatograph (Thermo Scientific, Sunnyvale, CA, USA) with an AccucoreTM aQ analytical column (150 mm × 2.1 mm; i.d. 2.6 µm; Thermo Scientific, San Jose, CA, USA) coupled with a Q-ExactiveTM high resolution tandem mass spectrometer (Thermo Scientific, Bremen, Germany) was followed^30^.

For validation of the method and verifying the performance characteristics (recoveries, repeatability, and LOQs), the internal reference sample of milk thistle-based dietary supplements, containing a very low or zero concentration of the contaminants were used. The external matrix-matched calibration batch with standards at levels of 0.05, 0.5, 1, 2, 5, 10, 25, 50, 100, and 200 μg/L (corresponding to 0.5–2,000 μg/kg) was prepared from the reference sample extract. The repeatability of the method, expressed as a relative standard deviation (RSD), was assessed by the analysis of “spikes” (*i.e*., a “blank” matrix fortified by the standard mixture at 500 μg/kg before extraction) in seven replicates, which ranged between 0.3 to 11.5%, depending on the particular analyte. The method for recoveries were estimated as the percentage ratio between the “determined” to “real” concentration of a spike, and for most of analytes ranged between 68 to 109%. The LOQs were estimated as the lowest concentration level of matrix-matched calibration standard providing a stable signal over four consecutive days, and ranged between 0.5 and 1,250 μg/kg, depending on the particular analyte (see Suppl. Table S2). For quantification of the analytes’ concentrations, an external matrix matched calibration was performed, and a correction of the results for particular analytes recoveries was performed. For quantification, the standard addition method utilized in our previous study^30^ was used.

### Determination of microbiological purity of the dietary supplements (isolation and determination of microorganisms)

Vials containing the capsules were surface sterilized with ethanol and aseptically opened in a flow box. Approximately 0.2 g of powder from the capsule was poured onto the agar plate surface. The experiment was done in triplicate in glass Petri dishes (average diameter 19 cm). In order to isolate the fungi, dichloran-glycerol (DG18), agar base (Oxoid CZ), and potato dextrose agar plates (Oxoid CZ) were used. LB broth agar was used for bacteria isolation. The plates were incubated for 7 days at 28 °C. After, the individual colonies were counted and analyzed.

The bacteria were identified directly from the colonies by MALDI TOF mass spectrometry. The unique molecular fingerprints of individual colonies were measured with an Autoflex Speed MALDI-TOF mass spectrometer using the direct transfer protocol recommended by the manufacturer (Bruker Daltonics, Billerica, MA, USA). The identification was performed by a MALDI Biotyper 3.1 (Bruker Daltonics) equipped with database version 4.0.0.1 that contains the fingerprints of 5,627 microorganisms.

The fungal DNA was obtained from lysed cells by a routine process. A single colony was suspended in 100 µl of deionized water by vortexing. After centrifugation (1 min, 10,000 × g) the pellet was re-suspended in lysis buffer (50 mM sodium phosphate, pH 7.4, 1 mM EDTA, 5% glycerol) and incubated for 30 min at 85 °C. Aliquots (0.2 µL) were used as DNA templates for PCR in a total reaction volume of 12.5 µL, and the procedures were performed according to the manufacturer´s recommendations (Kapa HiFi Hot Start Polymerase, Elisabeth Pharmacon, Surrey, UK). The fungi were identified by sequencing their internal transcribed spacer (ITS) fragment (550 bp) using the common primers ITS1 (TCCGTAGGTGAACCTGCGG) and ITS4 (TCCTCCGCTTATTGATATGC). Purification of the PCR products was performed with a Zymoclean Gel DNA Recovery Kit (Zymo Research, Irvine, CA, USA). The purified PCR products were sequenced by GATC Biotech AG (Cologne, Germany). The sequence comparisons were done by utilizing the pairwise sequence alignments against the ITS reference database in the ISHAM ITS Database (http://its.mycologylab.org/).

## Results

### Silymarin composition

The composition of silymarin was analyzed for 26 preparations (from eight US and seven Czech manufacturers) (Table 1). In some cases, different batches of identical preparations were purchased within two years in different US States.

### Variability in maximal daily dose for individual milk thistle preparations recommended by manufacturers

**Figure 1:**
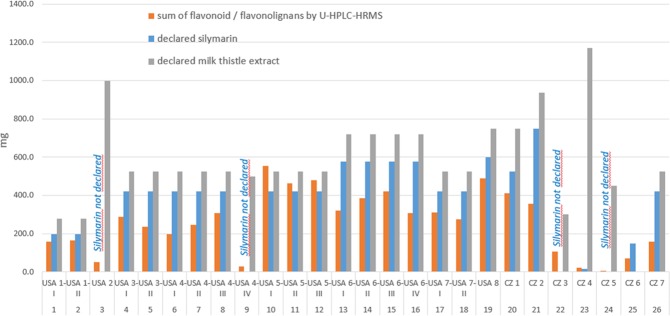
The silymarin complex (mg) in the maximum daily dose of preparation recommended by individual producers. The recommended maximum daily dose was based on determination of the weight of internal content of the capsule and the number of capsules recommended by producers per day. The concentrations of individual flavonoids/flavonolignans in mg/g are presented in Suppl. Table S3. For sample description see Table 1.

Large variability in the maximal daily dose of silymarin/milk thistle extract recommended by individual producers exists in both the US and Czech Republic (Fig. 1). None of the manufacturers defined individual flavonoid/flavonolignan composition; with most of them instead using the general term “silymarin content” covering all the biologically active components. Four manufacturers (out of 15) used an even the more general term “milk thistle extract” to describe the active constituents.

The maximal daily dose for silymarin, i.e., the declared silymarin content in the maximal dosage of the dietary supplements, varied from 196 to 600 mg per day in the US (two manufacturers did not declare silymarin content, but only amount of milk thistle extract), compared with 18 to 500 mg per day in the Czech Republic (two manufacturers did not declare silymarin content, but only amount of milk thistle extract).

The maximal daily dose for milk thistle extract varied from 280 to 1,000 mg per day in the US, compared with 175 to 1,170 mg per day in the Czech Republic (Fig. 1).

### Variability in flavonoid/flavonolignan content in individual milk thistle preparations from declarations of silymarin as given by manufacturers

Because the terminology in this specific field is rather complicated and not defined by legislation, the term “silymarin” is used by producers in a rather casual manner, and usually includes all (major) flavonolignans. When considering the flavonoids/flavonolignans as the major silymarin components, their total concentration was found to range from 35–125% of the declared “silymarin”. Flavonoid/flavonolignan concentrations comprising less than 50% of the declared silymarin content were detected in samples Nos. 6 (USA 4-I), 21 (CZ2), 25 (CZ6), and 26 (CZ7) (Fig. 1). On the other hand, a higher content of flavonoids/flavonolignans than that declared for silymarin was observed in samples Nos. 10-12 (USA 5 I-III), and 23 (CZ4).

We are aware that the direct comparison, described above, is not entirely accurate due to the possible presence of the undefined polymeric bioactive fraction^5^. However, the large discrepancies observed between the declared and experimentally determined “silymarin” content highlight the obvious problems with silymarin standardization, even with various batches of the same preparations produced by the same manufacturers, e.g., for samples No. 6–9 (USA 4 I-IV), the maximum difference between the samples in flavonoid/flavonolignan content was 277 mg of the product’s recommended daily dose (Fig. 1). It is also important to note that the content of less frequent flavonolignans such as taxifolin or 2,3-dehydosilybin A an B differed substantially across analyzed samples (Suppl. Table S3).

### Variability in individual flavonoid/flavonolignan composition across tested milk thistle preparations

**Figure 2:**
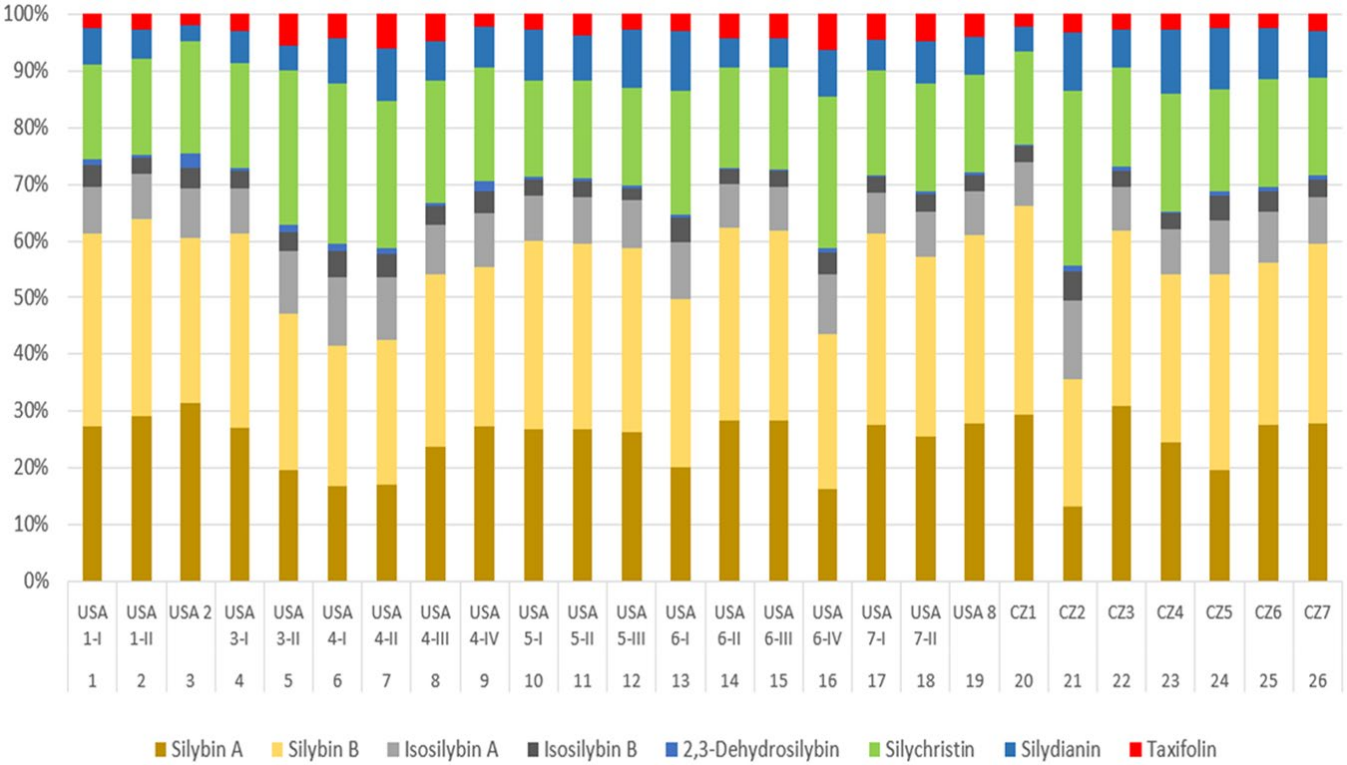
Composition of flavonoids/flavonolignans in individual milk thistle preparations. 100% is the summary concentration of all flavonoids/flavonolignans present in sample. For sample description see Table 1.

Marked differences in the content of individual flavonoids/flavonolignans were found across different milk thistle preparations, even within different batches by the same manufacturers (Fig. 2). This can be demonstrated on silybin A and silybin B, the most abundant flavonolignans in the milk thistle preparations. As can be seen from Fig. 2, the sum of silybin A and B varied from 36% to 66% between samples (where the sum of all flavonoids/flavonolignans was considered as 100%). The greatest variability within different batches by the same producer was for USA producer No. 6 (Fig. 2), the minimum and maximum values were 44% and 62%, respectively.

### Safety issues

To perform a comprehensive assessment of the biological safety of this dietary supplement group, the multi-analyte method in total targeting 55 toxic secondary fungal metabolites, 11 plant alkaloids, and 323 pesticide residues was employed for analysis of all 26 milk thistle preparations representing both the U.S. and Czech markets.

### Mycotoxins

**Figure 3:**
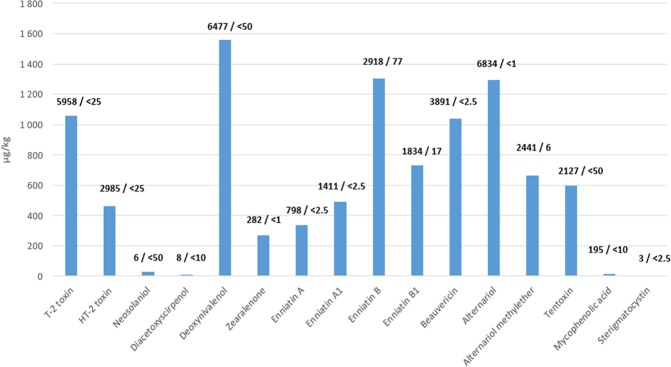
Concentrations of mycotoxins determined in individual milk thistle preparations. Numbers above the columns indicate the maximum/minimum concentration value.

Generally, the most frequent mycotoxins found in the milk thistle-based preparations were the *Fusarium* mycotoxins; *i.e*., type A trichothecenes (HT-2 and T2 toxins occurred in 92% and 96% of the samples, respectively), type B trichothecene deoxynivalenol occurred in 81% of the samples, zearalenone occurred in 89%, cyclic depsipeptidic mycotoxins as enniatines and beauvericin occurring, in 96–100%, and mycotoxins (produced by *Alternaria* fungi) had an occurrence frequency of 96–100% (Table 2, Fig. 3).

**Table 2:** Concentrations of mycotoxins in investigated milk thistle preparations.

Sample No.	Sampling year	Sample code (country of origin, brand- batch No)	Mycotoxin concentrations (µg/kg)															
			T-2 toxin	HT-2 toxin	Neoso-laniol	Diace-toxy-scirpenol	Deoxyni-valenol	Zeara-lenone	Ennia-tine A	Ennia-tine A1	Ennia-tine B	Ennia-tine B1	Beauve-ricine	Alter-nariol	Alter-nariol-methyl-ether	Ten-toxin	Myco-phenolic acid	Steri-gmato-cystin
**1**	2016	**USA 1-I**	**1,064**	**421**	<50	<10	**695**	**214**	**798**	**1,411**	**2,720**	**1,834**	**3,030**	**1,840**	**1,485**	**1,086**	<10	<2.5
**2**	2017	**USA 1-II**	**363**	**138**	<50	<10	**219**	**84**	**252**	**331**	**903**	**508**	**464**	**1,334**	**763**	**527**	<10	<2.5
**3**	2016	**USA 2**	**293**	**89**	<50	<10	<50	**21**	**501**	**628**	**1,127**	**855**	**436**	**140**	**50**	**309**	<10	<2.5
**4**	2016	**USA 3-I**	**1,758**	**553**	**41**	**59**	**4,124**	**205**	**476**	**567**	**1,297**	**865**	**1,442**	**1,467**	**623**	**779**	<10	<2.5
**5**	2017	**USA 3-II**	**311**	**72**	<50	<10	**885**	**63**	**325**	**375**	**1,900**	**786**	**276**	**572**	**275**	**555**	<10	<2.5
**6**	2016	**USA 4-I**	**2,551**	**750**	60	<10	**2,631**	**248**	**579**	**829**	**1,760**	**1,060**	**2,116**	**2,074**	**1,264**	**706**	<10	<2.5
**7**	2017	**USA 4-II**	**412**	**126**	<50	<10	**1,186**	**100**	**190**	**227**	**871**	**406**	**338**	**510**	**256**	**364**	<10	<2.5
**8**	2017	**USA 4-III**	**517**	**153**	<50	<10	**722**	**48**	**126**	**143**	**329**	**218**	**453**	**562**	**289**	**385**	<10	<2.5
**9**	2017	**USA 4-IV**^**b**^	**275**	**91**	<50	<10	**112**	**25**	**238**	**245**	**869**	**473**	**201**	**228**	**63**	**344**	<10	<2.5
**10**	2016	**USA 5-I**	**2,176**	**659**	<50	**46**	**4,576**	**274**	**603**	**877**	**1,752**	**1,099**	**2,388**	**1,823**	**1,322**	**986**	<10	<2.5
**11**	2017	**USA 5-II**	**794**	**237**	<50	<10	**3,191**	**106**	**588**	**852**	**2,880**	**1,230**	**962**	**554**	**462**	**521**	<10	<2.5
**12**	2017	**USA 5-III**	**672**	**249**	<50	<10	**3,582**	**260**	**540**	**698**	**2,580**	**1,198**	**733**	**1,690**	**919**	**696**	<10	3
**13**	2016	**USA 6-I**	**2,735**	**996**	**66**	**55**	**4,395**	**253**	**739**	**1,200**	**2,440**	**1,427**	**3,310**	**1,973**	**1,623**	**1,086**	<10	<2.5
**14**	2017	**USA 6-II**	**840**	**310**	<50	<10	<50	**60**	**165**	**195**	**611**	**294**	**590**	**452**	**273**	**328**	<10	<2.5
**15**	2017	**USA 6-III**	**856**	**295**	<50	<10	<50	**56**	**161**	**194**	**573**	**281**	**599**	**437**	**250**	**315**	<10	<2.5
**16**	2017	**USA 6-IV**	**369**	**179**	<50	<10	**1 162**	**108**	**263**	**343**	**1 363**	**572**	**504**	**469**	**294**	**464**	<10	<2.5
**17**	2017	**USA 7-I**	**193**	**40**	<50	<10	**557**	**42**	**88**	**95**	**263**	**159**	**207**	**277**	**127**	**286**	<10	<2.5
**18**	2017	**USA 7-II**	**527**	**182**	<50	<10	**731**	**86**	**389**	**538**	**1,255**	**848**	**506**	**607**	**270**	**392**	<10	<2.5
**19**	2017	**USA 8**	**843**	**236**	<50	<10	**1,517**	**144**	**416**	**593**	**1,405**	**844**	**676**	**749**	**385**	**488**	<10	<2.5
**20**	2016	**CZ 1**	**801**	**907**	<50	**36**	**2,363**	**189**	**225**	**463**	**1,738**	**819**	**1,161**	**4,145**	**1,648**	**1,509**	**195**	<2.5
**21**	2016	**CZ 2**	**5,958**	**2,985**	<50	<10	**6,477**	**282**	**722**	**1,142**	**2,918**	**1,822**	**3,891**	**6,834**	**2,441**	**2,127**	<10	<2.5
**22**	2016	**CZ 3**	**812**	**565**	<50	<10	**418**	**57**	**83**	**196**	**651**	**392**	**790**	**2,002**	**694**	**404**	<10	<2.5
**23**	2016	**CZ 4**	<25	<25	<50	<10	<50	<1	<2.5	**7**	**156**	**78**	<2.5	<1	**6**	<50	<10	<2.5
**24**	2016	**CZ 5**	<25	**100**	<50	<10	<50	<1	**14**	<2.5	**77**	**17**	**87**	**58**	**103**	**23**	**35**	<2.5
**25**	2016	**CZ 6**	**1,328**	**974**	<50	<10	**106**	<1	**170**	**251**	**732**	**400**	**823**	**920**	**524**	**325**	<10	<2.5
**26**	2016	**CZ 7**	**1,039**	**651**	<50	<10	**772**	**76**	**129**	**304**	**777**	**505**	**976**	**1,964**	**807**	**534**	<10	<2.5

The mean concentrations of *Fusarium* mycotoxins trichothecenes, enniatins, beauvericin, and *Alternaria* mycotoxins were typically on the order of single digits of mg/kg (for details see Table 2 and Fig. 3).

Of the mycotoxins detected in the samples investigated, the EU legislation places a maximum limit for only HT-2 and T-2 toxins, deoxynivalenol, and zearalenone; whereas in U.S. legislation, only deoxynivalenol is included. Furthermore, in neither the EU nor the US are herbal-based dietary supplements included as a separately assessed food group. To evaluate the severity of contamination, the concentrations determined in our study were compared with those having EU maximum limits for other food commodities (Suppl. Table S4). Surprisingly, it was found that they often dramatically exceeded even the highest maximum limits. For example, the maximum allowed concentration level of HT-2 and T-2 toxins in cereals intended for human consumption is 200 μg/kg^31^; however, the sum of HT-2 and T-2 toxins in 92% of the investigated milk thistle-based preparations exceeded this value by up to 19× (Table 2 and Fig. 3).

The risks associated with the occurrence of mycotoxins were assessed by a comparison of the daily mycotoxin exposures calculated from the recommended dosage of the particular milk thistle preparation against the tolerable daily intake (TDI) for mycotoxins established by the Scientific Committee on Food of the European Food Safety Authority^32^. Unfortunately, as TDI values have not yet been set for all of the mycotoxins; thus, from the mycotoxins present in our samples only the sum of the HT-2 and T-2 toxins (deoxynivalenol and zearalenone) could be assessed by this method. Nevertheless, the most critical situation was observed for the sum of HT-2 and T-2 toxins, for which the TDI value was set very low, 0.1 µg/kg b.w. (Table 3). When considering a 70 kg man, at least one quarter of the TDI was fulfilled by almost 50% of the samples; and in one sample, the TDI was exceeded by approximately 3× (Table 3).

**Table 3:** Daily intake of mycotoxins expressed as percentage of tolerable daily intake after taking the recommended dosage of individual milk thistle preparations.

Sample code (country of origin, brand-batch No)	Recommended dosage (No. of capsules)	Percentage of mycotoxin tolerable daily intake		
		(T-2 toxin + HT-2 toxin)*	Deoxynivalenol**	Zearalenone***
USA 1-I	2	15	0.7	0.8
USA 1-II	2	5	0.2	0.3
USA 2	4	12	0.0	0.3
USA 3-I	3	52	9.4	1.9
USA 3-II	3	8	2.0	0.6
USA 4-I	3	76	6.1	2.3
USA 4-II	3	12	2.6	0.9
USA 4-III	3	15	1.7	0.4
USA 4-IV	2	6	0.2	0.2
USA 5-I	3	54	8.7	2.1
USA 5-II	3	19	5.8	0.8
USA 5-III	3	17	6.5	1.9
USA 6-I	6	78	9.2	2.1
USA 6-II	6	25	0.0	0.5
USA 6-III	6	25	0.0	0.5
USA 6-IV	6	11	2.4	0.9
USA 7-I	3	5	1.2	0.4
USA 7-II	3	16	1.7	0.8
USA 8	3	31	4.3	1.7
CZ 1	3	31	4.4	0.3
CZ 2	3	318	23.0	1.0
CZ 3	3	28	0.9	0.1
CZ 4	3	0	0.0	0.0
CZ 5	3	2	0.0	0.0
CZ 6	3	44	0.2	0.0
CZ 7	3	35	1.6	0.2

Fulfillment of mycotoxin TDI for a 70 kg man, data expressed in %.

*TDI for (HT-2 toxin + T2 toxin) = 0.1 µg/kg b.wt; **TDI for deoxynivalenol = 1 µg/kg b.wt; ***TDI for zearalenone = 0.25 µg/kg b.wt. (37).

TDI, tolerable daily intake.

**Figure 4:**
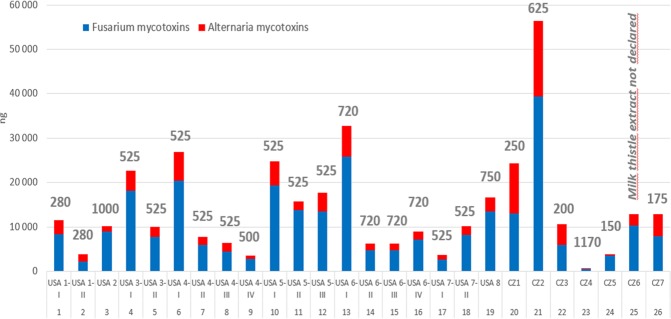
Total mycotoxin content in the maximum dietary supplement dosage recommended by producers. Grey numbers above the columns indicate mg of milk thistle extract in the maximum dosage of capsules. For sample description see Table 1.

Although a linear relationship between the mycotoxin load and the amount of milk thistle extract in the individual milk thistle preparations might be expected, this was not the fact (Fig. 4), indicating a large variability in the quality of milk thistle (*Silybum marianum*) plants used by individual manufacturers.

### Plant alkaloids

Out of eleven plant alkaloids tested, none were detected in the investigated samples, although the detection limits were very low (in order of single units in ppb, data not shown). Although some studies had published that the concentrations of plant alkaloids in herbal-based dietary supplements were the result of weed contamination of the herbal materials^33,34^, this was not confirmed in the investigated milk thistle preparations.

### Pesticide residues

Of the 323 compounds investigated, only five were detected and quantified (*i.e*., the insecticides pirimiphos-methyl, malathion, chlorpyrifos; and the fungicide carbendazim), as well as piperonyl butoxide (a component of pesticide formulations enhancing the potency of certain pesticides) (Table 4). To assess the severity of contamination by these pesticide residues, the detected concentrations were compared with the EU maximum residue levels (MRLs) set by Regulation 396/2005/EC (and available online in a complex EU pesticide database (http://ec.europa.eu/food/plant/pesticides/eu-pesticides-database/public); accessed on 27^th^ Sept, 2018). Similarly as for mycotoxins, the MRLs for pesticides are not specifically defined for milk thistle-based dietary supplements. Nevertheless, when comparing the concentrations determined with the MRLs for herbs and edible flowers (which are 20, 20, 50, and 100 µg/kg for pirimiphos-methyl, malathion, chlorpyrifos, and carbendazim, respectively - as defined in the sources stated above), these maximal limits were exceeded in three cases (specifically in samples No. 13 and 21 for pirimiphos-methyl, malathion, and carbendazim). Taking into account that the target group of people taking these preparations are mostly patients with liver diseases, the relationship of pesticide concentrations to the strictest MRLs (10 µg/kg as defined for babies and infants) could become even more meaningful. In this case, all of the pesticide concentrations detected in the samples would exceed the “safe” limit for individual compounds (not taking into account the possible additive effect of “cocktails” of several toxic compounds).

**Table 4:** Concentrations of pesticide residues in investigated milk thistle preparations.

Sample No.	Sampling year	Sample code (country of origin, brand-batch No)	Pesticide concentrations (µg/kg)				
			Pirimiphos-methyl	Malathion	Chlorpyrifos	Carbendazim	Piperonyl butoxide
1	2016	**USA 1-I**	<1	<2.5	<1	<2.5	<10
2	2017	**USA 1-II**	<1	<2.5	<1	**24.0**^**a**^	<10
3	2016	**USA 2**	<1	<2.5	<1	<2.5	<10
4	2016	**USA 3-I**	<1	<2.5	<1	<2.5	<10
5	2017	**USA 3-II**	<1	<2.5	<1	<2.5	<10
6	2016	**USA 4-I**	<1	<2.5	<1	<2.5	**35**^**a**^
7	2017	**USA 4-II**	<1	<2.5	<1	**12.6**^**a**^	<10
8	2017	**USA 4-III**	<1	<2.5	<1	**29.0**^**a**^	<10
9	2017	**USA 4-IV**	<1	<2.5	<1	<2.5	<10
10	2016	**USA 5-I**	<1	<2.5	<1	<2.5	<10
11	2017	**USA 5-II**	<1	<2.5	<1	**16.8**^**a**^	<10
12	2017	**USA 5-III**	<1	<2.5	<1	<2.5	<10
13	2016	**USA 6-I**	<1	**27.5**^**a,b**^	**38.2**^**a**^	**118**^**a,b**^	<10
14	2017	**USA 6-II**	<1	<2.5	<1	<2.5	<10
15	2017	**USA 6-III**	<1	<2.5	<1	<2.5	<10
16	2017	**USA 6-IV**	<1	<2.5	<1	**16.2**^**a**^	<10
17	2017	**USA 7-I**	<1	<2.5	<1	<2.5	<10
18	2017	**USA 7-II**	<1	<2.5	<1	<2.5	<10
19	2017	**USA 8**	<1	<2.5	<1	<2.5	<10
20	2016	**CZ 1**	**14.1**^**a**^	<2.5	<1	<2.5	<10
21	2016	**CZ 2**	**28.9**^**a,b**^	<2.5	<1	<2.5	<10
22	2016	**CZ 3**	**6.7**^**a**^	<2.5	<1	<2.5	<10
23	2016	**CZ 4**	<1	<2.5	<1	<2.5	<10
24	2016	**CZ 5**	<1	<2.5	<1	<2.5	<10
25	2016	**CZ 6**	<1	<2.5	<1	<2.5	<10
26	2016	**CZ 7**	<1	<2.5	<1	<2.5	<10

^a^Concentration of pesticide exceeded the MRL value for babies and infants.

^b^Concentration of pesticide exceeded the MRL value for herbs and edible flowers.

### Microbiological analysis of the investigated milk thistle preparations

Microbial contamination of the milk thistle preparations analyzed was found to be extensive (Table 5, Suppl. Table S5). In total, 37 microbial species belonging to 18 genera were identified, with *Bacillus* and *Paenibacillus* being the principal bacteria detected in 42% of samples. Both genera are ubiquitous in the environment, especially in soils, often on plants (as endophytes), forming an important component of the microrhizome. Other bacterial genera present in the samples were biofilm-forming *Staphylococcus*, *Pseudomonas*, and *Escherichia*. As concerns fungi, *Aspergillus* was the main genera detected in 58% of samples.

## Discussion

The use of herbal-based dietary supplements by the general population for their potential beneficiary health effects has steadily increasing over recent decades despite their often unproven efficacy^1,2,35,36^. One reason for controversy about the clinical efficacy of many herbal-based dietary supplements is the lack of standardization in the herbal-based dietary supplements preparation process^2^. This also seems to be true for milk thistle supplements, which in some studies have appeared to be effective^5,7,9^, while other studies did not confirm these observations^2,8,10,11^. In this respect, it should be noted that analyzed silymarin preparations differed substantially in the content of some minor flavonolignans, such as 2,3-dehydrosilybins A and B (Suppl. Table S3), known to be biologically much more potent^37,38^. There are numerous factors affecting the composition of milk thistle preparations^13^, which certainly may account for the large variability of silymarin complex compositions observed in our current study. These factors include: *e.g*., the variety of the plants used, climatic conditions during plant growth, seasonal and geographic conditions, and/or methods of extraction of the silymarin flavonoids/flavonolignans^13^. The maintenance of Good Manufacturing Practices is also of significant importance, often being violated during production of these uncontrolled over-the-counter preparations^2^.

Our results showed very different levels of flavonoids/flavonolignans content across the food components tested (which is in line with the results reported in only two previous studies)^39,40^. It also should be noted that our results did not show any systematic differences in silymarin compositions between those sampled in the US and the Czech Republic. It should be also noted, however, that different batches of the same brand and producer did show differences in silymarin composition, likely due to the variable quality of the milk thistle being purchased by the producing companies.

Apart from the problem of variability in silymarin complex composition, health issues related to contaminating bioactive compounds become important. Together with explosive trends in the use of herbal-based dietary supplements in the general population, the increased incidence of herbal-based dietary supplements-induced liver injury is being recognized as a clinical problem^41,42^. Although most of these toxic liver effects seem to be idiosyncratic^42^, the possible harmful effects of contaminating biological factors are not being considered seriously. Among those factors, mycotoxins are a largely ignored global health threat; their potential harm to human health, wide-spread occurrence in the human food chain, missing or poorly defined legislation, as well as the lack of reliable analytical and diagnostic tools in natural products research to prevent general population exposures^43,44^. In our study, mycotoxins were present in the majority of the investigated milk thistle preparations, often in potentially dangerous concentrations. These observations seem to have important clinical impacts, taking into account the fact that the milk thistle preparations containing immunotoxic, genotoxic, and hepatotoxic mycotoxins are primarily taken by patients suffering from liver diseases^45^. In addition, dietary supplements are certainly not the only source of mycotoxins, which ubiquitously occur in many food sources. Considering the high number and concentrations of mycotoxins co-occurring in the individual milk thistle preparations, the toxicological impacts might be enormous. As is known from many toxicological studies, not only the absolute concentration of any single mycotoxin can represent a problem, but also their co-occurrence in mixtures (‘cocktails’) could lead to significant health risks^46–48^. Some reports have revealed the additive or synergistic toxicological effects of particular mycotoxin combinations, demonstrating that the simultaneous presence of low doses of mycotoxin mixtures in food commodities and diet may be more toxic than is predicted from the individual mycotoxins alone^49^. In our study, the most frequent mycotoxin combinations (*i.e*., HT2 + T2 + DON + ZEA + enniatins + beauvericin + AOH + AME + TEN) were detected in as many as 77% of the samples, which is in good agreement with our previously published data^24^. It is also important to stress that the high hepatotoxicity potential of enniatins and beauvericin found in cereals was recently reported in a Danish study^50^, which again raises the question of how preparations with silymarin flavonolignans could act in a hepatoprotective manner when simultaneously contaminated with potentially hepatotoxic mycotoxins.

Another problem seems to be the presence of substantial amounts of pesticides, which certainly may affect the health of consumers. Although valid scientific data are largely missing, it is likely that pesticides contaminating milk thistle preparations may act against the potential hepatoprotective effects of silymarin flavonolignans. For instance, chlorpyrifos, one of the pesticides detected in the investigated milk thistle preparations was reported to be hepato- and nephrotoxic in orally-exposed mice^51^.

Further, the microbial contamination of milk thistle preparations should considered with caution, especially the detection of biofilm-forming bacteria of the *Staphylococcus*, *Pseudomonas*, and *Escherichia* genus, which may cause serious diseases, especially in immune-suppressed persons^52^. A major fungal genus present in 60% of the samples was *Aspergillus*, and even though mycotoxin-producing strains were identified in some samples, the corresponding mycotoxins were not present. This indicates that the fungi did not represent the original microbiota of the plant, which in fact could hardly survive the two-step extraction (the recommended procedure according to the European Pharmacopoeia: hexane for 6 h and methanol for 5 h)^53^ and following spray drying used by the manufacturers during the silymarin preparations industrial production. The secondary fungal contamination of the samples probably occurred during industrial extract powder handling and processing, encapsulation, packaging and further steps. The control of manufacturing processes leading to minimize end-product (supplement) contamination is the crucial step of Good Manufacturing Practices (GMPs)^54^. Improper handling of extract powder during distribution between the extract-producing company and supplements-producing company can lead to secondary microbial contamination as well as water, air or devices contamination of production facilities of the supplement manufacturer^55^. It is worth to notice that the inappropriate (home) storage conditions, especially humidity and temperature, can affect the viability of surviving fungi and support their growth, which may result in mycotoxin production and possibly extend the mycotoxin spectrum^56^. The microbial quality of some samples was of alarmingly poor microbial quality. Especially in one sample (USA 6-I), the fungal contamination exceeded the European limit for orally administered plant extracts by 1.3x^57^. Again, these dietary supplements are usually recommended to immune-compromised patients, and thus the risk of infection by opportunistic pathogens found in the samples may jeopardize their health conditions.

In conclusion, our results strongly indicate the need for strict controls over the composition, biological contaminants, as well as microbiological purity of commercial silymarin extracts used for the treatment of liver disease patients. Poor definitions of these preparations together with contamination by biologically active substances may not only account for the inconsistency of clinical observations, but may also be responsible for possible herbal-based dietary supplements-induced liver injury.

## Supplementary information

Dataset 1

## Data Availability

All the detailed data are available on request from the corresponding author (M.Z.).
