# Supplementary material for: Poor chemical and microbiological quality of the commercial milk thistle-based dietary supplements may account for their reported unsatisfactory and non-reproducible clinical outcomes
Source: Sci Rep. 2019 Jul 31;9:11118. doi: 10.1038/s41598-019-47250-0 (PMC6668463; doi:10.1038/s41598-019-47250-0)

**Poor chemical and microbiological quality of the commercial milk thistle-based dietary supplements may account for their reported unsatisfactory** **and non-reproducible clinical outcomes**

**Marie Fenclova^1^, Alena Novakova^1^, Jitka Viktorova^2^, Petra Jonatova^1^, Zbynek Dzuman^1^, Tomas Ruml^2^, Vladimir Kren^3^, Jana Hajslova^1^, Libor Vitek^4^, Milena Stranska-Zachariasova^1^**

^1^Department of Food Analysis and Nutrition, University of Chemistry and Technology, Technicka 3, 16628 Prague 6,

^2^Department of Biochemistry and Microbiology, University of Chemistry and Technology, Technicka 3, 16628 Prague 6,

^3^Laboratory of Biotransformation, Institute of Microbiology of the Czech Academy of Sciences; Vídeňská 1082, 14000, Prague 6

**^4^**Institute of Medical Biochemistry and Laboratory Diagnostics and 4^th^ Department of Internal Medicine, 1^st^ Faculty of Medicine, Charles University, Katerinska 32, 12108, Prague 2,

(All Czech Republic)

**Table S1. Complete list of target analytes (mycotoxins, plant alkaloids and pesticides)**

| **Mycotoxins** | 3-acetyldeoxynivalenol, 15-acetyldeoxynivalenol, aflatoxin B1, aflatoxin B2, aflatoxin G1, aflatoxin G2, agroclavine, alpha-zearalenol, alternariol, alternariol-methylether, beauvericin, beta-zearalenol, citrinin, cyclopiazonic acid, deoxynivalenol, deoxynivalenol-3-glucoside, diacetoxyscirpenol, enniatin A, enniatin A1, enniatin B, enniatin B1, ergocornine, ergocorninine, ergocristine, ergocristinine, ergocryptine, ergocryptinine, ergometrine, ergosine, ergosinine, ergotamine, ergotaminine, fumonisin B1, fumonisin B2, fumonisin B3, fusarenon X, gliotoxin, HT-2 toxin, meleagrin, mycophenolic acid, neosolaniol, nivalenol, ochratoxin A, patulin, paxilline, penicillic acid, penitrem A, phomopsin A, roquefortine C, stachybotrylactam, sterigmatocystin, T-2 toxin, tentoxin, verrucarol, verruculogen, zearalenone |
| --- | --- |
| **Pyrrolizidine alkaloids** | heliotrine, lycopsamine, monocrotaline, monocrotaline N-oxide, retrorsine, retrorsine N-oxide, senecionine, senecionine N-oxide, seneciphylline, seneciphylline N-oxide, senkirkine |
| **Pesticides** | 2,4,5-T, 2,4-D, 2,4-DB, 2-NOA, 4-CPA, acephate, acetamiprid, acetochlor, acrinathrin, alachlor, aldicarb, aldicarb sulfone, aldicarb sulfoxide, ametryn, atrazine, avermectin-b1a, azadirachtin, azinphos-ethyl, azinphos-methyl, azoxystrobin, benalaxyl, bendiocarb, bentazone, beta-cyfluthrin, bifenthrin, bitertanol, boscalid, bromacil, bromoxynil, bromuconazole, bupirimate, buprofezin, cadusafos, carbaryl, carbendazim, carbofuran, carbofuran-3-hydroxy, carbophenothion, chlorantraniliprole, chlorfenvinphos, chloridazon, chlorotoluron, chloroxuron, chlorpyrifos, chlorpyrifos-methyl, chlorsulfuron, cinerin I, cinerin II, clofentezine, clomazone, clothianidin, cyanazine, cyazofamid, cymoxanil, cypermethrin, cyproconazole, cyprodinil, DEET, deltamethrin, demeton-S-methyl, demeton-S-methylsulfone, desmedipham, desmethyl-pirimicarb, desmetryn, diazinon, dichlofluanid, dichlormid, dichlorprop, dichlorvos, diclofop-methyl, dicrotophos, diethofencarb, difenoconazole, diflubenzuron, diflufenican, dimethachlor, dimethenamide, dimethoate, dimethomorph, dimoxystrobin, diniconazole, disulfoton, disulfoton-sulfone, disulfoton-sulfoxide, diuron, DMSA, DMST, dodine (acetate), EPN, epoxiconazole, ethiofencarb, ethion, ethirimol, ethofumesate, ethoprophos, etofenprox, etrimfos, famoxadone, fenamiphos, fenamiphos sulphone, fenamiphos-sulfoxide, fenarimol, fenazaquin, fenbuconazole, fenbutatin-oxide, fenhexamid, fenoprop, fenoxaprop, fenoxaprop-ethyl, fenoxycarb, fenpropathrin, fenpropidin, fenpropimorph, fenpyroximate, fensulfothion, fenthion, fenthion-sulfone, fenthion-sulfoxide, fipronil, flonicamid, florasulam, fluacrypyrim, fluazifop, fluazifop-P-butyl, fluazinam, flucythrinate, fludioxonil, flufenacet, flufenoxuron, flumioxazin, fluopyram, fluoxastrobin, fluquinconazole, fluroxypyr, flusilazole, flutriafol, fomesafen, fonofos, foramsulfuron, formetanate hydrochlorid, formothion, fosthiazate, furathiocarb, haloxyfop, haloxyfop-2-ethoxyethyl, haloxyfop-methyl, heptenophos, hexaconazole, hexazinone, hexythiazox, imazalil, Imazamethabenz-methyl, imazamox, imazapyr, imazaquin, imazethapyr, imazosulfuron, imidacloprid, indoxacarb, iodosulfuron-methyl, ioxynil, iprovalicarb, isofenphos, isofenphos-methyl, isoprocarb, isoprothiolane, isoproturon, jasmolin I, jasmolin II, kresoxim-methyl, lambda-cyhalothrin, lenacil, linuron, lufenuron, malaoxon, malathion, mandipropamide, MCPA, MCPB, mecarbam, mecoprop, mefenpyr-diethyl, mepanipyrim, mepronil, metaflumizone, metalaxyl, metamitron, metazachlor, metconazole, methacriphos, methamidophos, methidathion, methiocarb, methiocarb-sulfone, methiocarb sulfoxide, metholcarb, methomyl, methoxyfenozide, metobromuron, metolachlor, metosulam, metoxuron, metribuzin, metsulfuron-methyl, mevinphos, monocrotophos, monolinuron, monuron, myclobutanil, naled, napropamide, neburon, nicosulfuron, norflurazone, omethoate, oxadixyl, oxamyl, oxydemeton-methyl, oxyfluorfen, paclobutrazol, penconazole, pencycuron, pendimethalin, permethrin, phenmedipham, phenothrin, phenthoate, phorate, phorate-sulfone, phorate-sulfoxide, phosalone, phosmet, phosphamidon, phoxim, picloram, picolinafen, picoxystrobin, inoxaden, piperonyl butoxide, pirimicarb, pirimiphos-ethyl, pirimiphos-methyl, prochloraz, profenofos, prometon, prometryn, propachlor, propamocarb, propaquizafop, propargite, propazine, propham, propiconazole, propoxur, propoxycarbazone, propyzamide, proquinazid, prosulfocarb, prothioconazole-desthio, prothiofos, pyraclostrobin, pyrazophos, pyrethrin I, pyrethrin II, pyridaben, pyridate, pyrifenox, pyrimethanil, pyriproxyfen, quinalphos, quinclorac, quinmerac, quinoclamine, quinoxyfen, quizalofop, quizalofop-p-ethyl, resmethrin, rimsulfuron, rotenone, simazine, simetryn, spinosyn A, spinosyn D, spirodiclofen, spiromesifen, spiroxamine, sulfosulfuron, sulfotep, tau-fluvalinate, tebuconazole, tebufenozide, tebufenpyrad, teflubenzuron, tepraloxydim, terbufos, terbufos-sulfone, terbufos-sulfoxide, terbuthylazine, terbutryn, tetraconazole, tetramethrin, thiabendazole, thiacloprid, thiamethoxam, thifensulfuron-methyl, thiodicarb, thiometon, thiophanate-methyl, tolclofos-methyl, tolylfluanid, triadimefon, triadimenol, triasulfuron, triazophos, trichlorfon, tricyclazole, trifloxystrobin, triflumuron, triforine, trinexapac ethyl, triticonazole, vamidothion, zoxamide |

**Table S2. Validation data for mycotoxins, alkaloids and pesticides analysed in individual milk thistle preparations**

| **No** | **MYCOTOXINS** | **Recovery** (%) | **Limit of quantification (LOQ)**  (µg/kg) | **Relative standard deviation (RSD)**  (%) |
| --- | --- | --- | --- | --- |
| 1 | 3+15-acetyl-DON | 78 | 12.5 | 6.0 |
| 2 | aflatoxin B1 | 80 | 0.5 | 2.7 |
| 3 | aflatoxin B2 | 84 | 2.5 | 4.4 |
| 4 | aflatoxin G1 | 82 | 0.5 | 6.2 |
| 5 | aflatoxin G2 | 86 | 0.5 | 7.8 |
| 6 | agroclavine | 98 | 0.5 | 5.5 |
| 7 | alpha-zearalenol | 87 | 12.5 | 5.8 |
| 8 | alternariol | 89 | 1 | 3.6 |
| 9 | alternariol-methylether | 84 | 1 | 6.2 |
| 10 | beauvericin | 98 | 2.5 | 5.7 |
| 11 | beta-zearalenol | 90 | 25 | 5.3 |
| 12 | citrinin | 79 | 50 | 8.4 |
| 13 | cyclopiazonic acid | 96 | 500 | 6.7 |
| 14 | deoxynivalenol | 88 | 50 | 1.7 |
| 15 | deoxynivalenol-3-glucoside | 48 | 250 | 7.8 |
| 16 | diacetoxyscirpenol | 80 | 10 | 6.9 |
| 17 | enniatin A | 87 | 2.5 | 7.5 |
| 18 | enniatin A1 | 85 | 2.5 | 3.3 |
| 19 | enniatin B | 82 | 2.5 | 3.6 |
| 20 | enniatin B1 | 79 | 2.5 | 1.3 |
| 21 | ergocornine | 91 | 12.5 | 8.7 |
| 22 | ergocorninine | 88 | 2.5 | 6.6 |
| 23 | ergocristine | 86 | 5 | 8.4 |
| 24 | ergocristinine | 84 | 2.5 | 7.2 |
| 25 | ergocryptine | 88 | 5 | 7.9 |
| 26 | ergocryptinine | 91 | 2.5 | 6.1 |
| 27 | ergometrine | 84 | 5 | 6.1 |
| 28 | ergosine | 84 | 2.5 | 6.1 |
| 29 | ergosinine | 93 | 2.5 | 4.4 |
| 30 | ergotamine | 99 | 2.5 | 2.8 |
| 31 | ergotaminine | 83 | 2.5 | 1.9 |
| 32 | fumonisin B1 | 76 | 50 | 4.6 |
| 33 | fumonisin B2 | 76 | 50 | 9.1 |
| 34 | fumonisin B3 | 88 | 50 | 5.4 |
| 35 | fusarenon X | 87 | 125 | 7.5 |
| 36 | gliotoxin | 84 | 125 | 8.7 |
| 37 | HT-2 toxin | 84 | 25 | 6.7 |
| 38 | meleagrin | 96 | 1.25 | 6.3 |
| 39 | mycophenolic acid | 79 | 10 | 5.0 |
| 40 | neosolaniol | 86 | 50 | 6.2 |
| 41 | nivalenol | 82 | 500 | 9.3 |
| 42 | ochratoxin A | 86 | 5 | 3.8 |
| 43 | patulin | - | 1250 | 6.9 |
| 44 | paxilline | 95 | 50 | 4.2 |
| 45 | penicillic acid | 92 | 125 | 2.7 |
| 46 | penitrem A | 99 | 125 | 3.5 |
| 47 | phomopsin A | 80 | 250 | 2.1 |
| 48 | roquefortine C | 98 | 2.5 | 5.9 |
| 49 | stachybotrylactam | 75 | 25 | 6.2 |
| 50 | sterigmatocystin | 87 | 2.5 | 6.5 |
| 51 | T-2 toxin | 80 | 25 | 6.2 |
| 52 | tentoxin | 68 | 50 | 4.1 |
| 53 | verrucarol | 73 | 125 | 3.3 |
| 54 | verruculogen | 106 | 250 | 5.9 |
| 55 | zearalenone | 86 | 1 | 5.5 |
| **No** | **PLANT ALKALOIDS** | **Recovery** (%) | **Limit of quantification (LOQ)**  (µg/kg) | **Relative standard deviation (RSD)**  (%) |
| 1 | heliotrine | 86 | 2.5 | 2.6 |
| 2 | lycopsamine | 68 | 12.5 | 2.1 |
| 3 | monocrotaline | 69 | 5 | 5.0 |
| 4 | monocrotaline N-oxide | 74 | 5 | 4.0 |
| 5 | retrorsine | 77 | 1.25 | 3.9 |
| 6 | retrorsine N-oxide | 69 | 1.25 | 3.2 |
| 7 | senecionine | 78 | 1.25 | 5.1 |
| 8 | senecionine N-oxide | 92 | 1.25 | 3.8 |
| 9 | seneciphylline | 88 | 0.5 | 5.1 |
| 10 | seneciphylline N-oxide | 90 | 0.5 | 6.6 |
| 11 | senkirkine | 83 | 0.5 | 5.2 |
| **No** | **PESTICIDES** | **Recovery** (%) | **Limit of quantification (LOQ)**  (µg/kg) | **Relative standard deviation (RSD)**  (%) |
| 1 | 2,4,5-T | 89 | 25 | 7.1 |
| 2 | 2,4-D | 92 | 12.5 | 7.0 |
| 3 | 2,4-DB | 87 | 250 | 8.1 |
| 4 | 2-NOA | 96 | 12.5 | 7.3 |
| 5 | 4-CPA | 95 | 12.5 | 7.9 |
| 6 | acephate | 84 | 12.5 | 8.1 |
| 7 | acetamiprid | 86 | 1.25 | 9.3 |
| 8 | acetochlor | 94 | 25 | 2.9 |
| 9 | acrinathrin | 89 | 25 | 2.3 |
| 10 | alachlor | 92 | 50 | 1.9 |
| 11 | aldicarb | 87 | 50 | 4.9 |
| 12 | aldicarb sulfone | 93 | 5 | 6.2 |
| 13 | aldicarb sulfoxide | 90 | 125 | 4.5 |
| 14 | ametryn | 93 | 0.5 | 3.1 |
| 15 | atrazine | 86 | 0.5 | 5.5 |
| 16 | avermectin-b1a | 87 | 25 | 6.3 |
| 17 | azadirachtin | 86 | 50 | 5.8 |
| 18 | azinphos-ethyl | 78 | 2.5 | 5.0 |
| 19 | azinphos-methyl | 86 | 2.5 | 8.1 |
| 20 | azoxystrobin | 87 | 0.5 | 4.2 |
| 21 | benalaxyl | 88 | 0.5 | 10.1 |
| 22 | bendiocarb | 88 | 1.25 | 3.3 |
| 23 | bentazone | 75 | 0.5 | 9.4 |
| 24 | beta-cyfluthrin | 81 | 500 | 8.7 |
| 25 | Bifenthrin | 80 | 50 | 7.6 |
| 26 | bitertanol | 86 | 5 | 11.2 |
| 27 | boscalid | 86 | 5 | 3.7 |
| 28 | bromacil | 81 | 5 | 7.2 |
| 29 | Bromoxynil | 81 | 12.5 | 3.8 |
| 30 | bromuconazole | 82 | 12.5 | 5.3 |
| 31 | bupirimate | 79 | 0.5 | 8.5 |
| 32 | buprofezin | 74 | 0.5 | 0.5 |
| 33 | cadusafos | 82 | 0.5 | 5.4 |
| 34 | carbaryl | 80 | 2.5 | 6.9 |
| 35 | carbendazim | 88 | 2.5 | 6.2 |
| 36 | carbofuran | 90 | 2.5 | 8.3 |
| 37 | carbofuran-3-hydroxy | 89 | 5 | 6.7 |
| 38 | carbophenothion | 93 | 250 | 10.7 |
| 39 | chlorantraniliprole | 87 | 2.5 | 5.5 |
| 40 | chlorfenvinphos | 92 | 2.5 | 5.5 |
| 41 | chloridazon | 84 | 0.5 | 2.9 |
| 42 | chlorotoluron | 86 | 0.5 | 2.1 |
| 43 | chloroxuron | 85 | 0.5 | 11.2 |
| 44 | chlorpyrifos | 92 | 1 | 10.2 |
| 45 | chlorpyrifos-methyl | 87 | 500 | 9.6 |
| 46 | chlorsulfuron | 90 | 2.5 | 6.0 |
| 47 | cinerin I | 87 | 50 | 7.0 |
| 48 | cinerin II | 84 | 250 | 6.2 |
| 49 | clofentezine | 88 | 125 | 7.1 |
| 50 | clomazone | 90 | 2.5 | 8.7 |
| 51 | clothianidin | 85 | 5 | 3.4 |
| 52 | cyanazine | 89 | 1.25 | 3.4 |
| 53 | cyazofamid | 77 | 5 | 4.7 |
| 54 | cymoxanil | 90 | 5 | 6.0 |
| 55 | cypermethrin | 86 | 50 | 10.3 |
| 56 | cyproconazole | 85 | 5 | 2.9 |
| 57 | cyprodinil | 83 | 0.5 | 0.3 |
| 58 | DEET | 92 | 0.5 | 1.7 |
| 59 | deltamethrin | 88 | 50 | 6.1 |
| 60 | demeton-S-methyl | 89 | 12.5 | 1.6 |
| 61 | demeton-S-methylsulfone | 94 | 0.5 | 6.9 |
| 62 | desmedipham | 89 | 0.5 | 8.2 |
| 63 | desmethyl-pirimicarb | 86 | 0.5 | 6.0 |
| 64 | desmetryn | 85 | 0.5 | 8.7 |
| 65 | diazinon | 87 | 0.5 | 8.9 |
| 66 | dichlofluanid | 82 | 12.5 | 9.5 |
| 67 | dichlormid | 89 | 12.5 | 3.2 |
| 68 | dichlorprop | 87 | 12.5 | 8.2 |
| 69 | dichlorvos | 109 | 12.5 | 7.5 |
| 70 | diclofop-methyl | 93 | 12.5 | 5.9 |
| 71 | dicrotophos | 91 | 0.5 | 4.0 |
| 72 | diethofencarb | 88 | 0.5 | 3.8 |
| 73 | difenoconazole | 84 | 5 | 2.1 |
| 74 | diflubenzuron | 86 | 5 | 1.6 |
| 75 | diflufenican | 87 | 5 | 9.1 |
| 76 | dimethachlor | 93 | 0.5 | 5.9 |
| 77 | dimethenamide | 91 | 1.25 | 1.7 |
| 78 | dimethoate | 92 | 0.5 | 3.2 |
| 79 | dimethomorph | 88 | 1.25 | 4.9 |
| 80 | dimoxystrobin | 90 | 0.5 | 0.4 |
| 81 | diniconazole | 91 | 5 | 6.3 |
| 82 | disulfoton | 79 | 500 | 11.2 |
| 83 | disulfoton-sulfone | 93 | 0.5 | 9.0 |
| 84 | disulfoton-sulfoxide | 96 | 0.5 | 4.5 |
| 85 | diuron | 91 | 0.5 | 5.0 |
| 86 | DMSA | 94 | 50 | 7.8 |
| 87 | DMST | 96 | 1.25 | 5.3 |
| 88 | dodine (acetate) | 96 | 0.5 | 6.5 |
| 89 | EPN | 90 | 0.5 | 4.9 |
| 90 | epoxiconazole | 93 | 2.5 | 7.1 |
| 91 | ethiofencarb | 73 | 2.5 | 1.4 |
| 92 | ethion | 88 | 2.5 | 4.0 |
| 93 | ethirimol | 79 | 0.5 | 7.9 |
| 94 | ethofumesate | 84 | 1.25 | 8.9 |
| 95 | ethoprophos | 86 | 2.5 | 1.0 |
| 96 | etofenprox | 78 | 2.5 | 6.4 |
| 97 | etrimfos | 84 | 2.5 | 7.2 |
| 98 | famoxadone | 81 | 25 | 2.2 |
| 99 | fenamiphos | 83 | 0.5 | 3.9 |
| 100 | fenamiphos sulphone | 87 | 0.5 | 6.7 |
| 101 | fenamiphos-sulfoxide | 92 | 0.5 | 7.1 |
| 102 | fenarimol | 84 | 12.5 | 10.7 |
| 103 | fenazaquin | 69 | 0.5 | 3.9 |
| 104 | fenbuconazole | 83 | 1.25 | 3.7 |
| 105 | fenbutatin-oxide | 70 | 12.5 | 6.0 |
| 106 | fenhexamid | 68 | 1.25 | 6.7 |
| 107 | fenoprop | 83 | 5 | 3.4 |
| 108 | fenoxaprop | 73 | 12.5 | 5.7 |
| 109 | fenoxaprop-ethyl | 79 | 0.5 | 5.6 |
| 110 | fenoxycarb | 71 | 0.5 | 4.6 |
| 111 | fenpropathrin | 73 | 50 | 7.9 |
| 112 | fenpropidin | 83 | 0.5 | 3.7 |
| 113 | fenpropimorph | 81 | 0.5 | 4.0 |
| 114 | fenpyroximate | 85 | 0.5 | 5.5 |
| 115 | fensulfothion | 88 | 0.5 | 6.4 |
| 116 | fenthion | 94 | 125 | 6.8 |
| 117 | fenthion-sulfone | 88 | 0.5 | 7.4 |
| 118 | fenthion-sulfoxide | 91 | 0.5 | 8.5 |
| 119 | fipronil | 94 | 50 | 3.1 |
| 120 | flonicamid | 93 | 12.5 | 7.5 |
| 121 | florasulam | 89 | 1.25 | 7.2 |
| 122 | fluacrypyrim | 89 | 0.5 | 3.5 |
| 123 | fluazifop | 86 | 2.5 | 2.8 |
| 124 | fluazifop-P-butyl | 89 | 0.5 | 5.7 |
| 125 | fluazinam | 89 | 0.5 | 2.8 |
| 126 | flucythrinate | 92 | 25 | 2.3 |
| 127 | fludioxonil | 88 | 0.5 | 2.1 |
| 128 | flufenacet | 91 | 0.5 | 8.6 |
| 129 | flufenoxuron | 88 | 5 | 8.9 |
| 130 | flumioxazin | 92 | 125 | 5.8 |
| 131 | fluopyram | 89 | 0.5 | 5.0 |
| 132 | fluoxastrobin | 89 | 0.5 | 5.2 |
| 133 | fluquinconazole | 90 | 12.5 | 3.5 |
| 134 | fluroxypyr | 86 | 500 | 3.5 |
| 135 | flusilazole | 86 | 5 | 8.9 |
| 136 | flutriafol | 85 | 2.5 | 3.9 |
| 137 | fomesafen | 87 | 2.5 | 8.9 |
| 138 | fonofos | 89 | 125 | 5.0 |
| 139 | foramsulfuron | 83 | 12.5 | 4.6 |
| 140 | formetanate hydrochlorid | 84 | 5 | 4.8 |
| 141 | formothion | 86 | 12.5 | 2.5 |
| 142 | fosthiazate | 90 | 0.5 | 4.9 |
| 143 | furathiocarb | 90 | 0.5 | 1.6 |
| 144 | haloxyfop | 87 | 50 | 7.4 |
| 145 | haloxyfop-2-ethoxyethyl | 90 | 1.25 | 7.7 |
| 146 | haloxyfop-methyl | 90 | 2.5 | 5.6 |
| 147 | heptenophos | 91 | 1.25 | 6.6 |
| 148 | hexaconazole | 86 | 2.5 | 2.5 |
| 149 | hexazinone | 89 | 1.25 | 4.0 |
| 150 | hexythiazox | 87 | 5 | 7.6 |
| 151 | imazalil | 87 | 0.5 | 3.1 |
| 152 | Imazamethabenz-methyl | 91 | 0.5 | 3.4 |
| 153 | imazamox | 93 | 0.5 | 6.9 |
| 154 | imazapyr | 90 | 0.5 | 7.1 |
| 155 | imazaquin | 87 | 0.5 | 8.8 |
| 156 | imazethapyr | 90 | 0.5 | 9.2 |
| 157 | imazosulfuron | 91 | 5 | 4.3 |
| 158 | imidacloprid | 91 | 5 | 4.5 |
| 159 | indoxacarb | 91 | 5 | 5.4 |
| 160 | iodosulfuron-methyl | 89 | 12.5 | 10.3 |
| 161 | ioxynil | 78 | 1.25 | 4.5 |
| 162 | iprovalicarb | 88 | 1.25 | 2.4 |
| 163 | isofenphos | 93 | 12.5 | 4.9 |
| 164 | isofenphos-methyl | 84 | 25 | 3.7 |
| 165 | isoprocarb | 94 | 2.5 | 8.1 |
| 166 | isoprothiolane | 91 | 0.5 | 9.8 |
| 167 | isoproturon | 85 | 0.5 | 2.1 |
| 168 | jasmolin I | 85 | 125 | 1.2 |
| 169 | jasmolin II | 88 | 125 | 7.8 |
| 170 | kresoxim-methyl | 88 | 1.25 | 6.1 |
| 171 | lambda-cyhalothrin | 81 | 50 | 4.3 |
| 172 | lenacil | 84 | 0.5 | 5.5 |
| 173 | linuron | 84 | 5 | 7.9 |
| 174 | lufenuron | 91 | 50 | 9.3 |
| 175 | malaoxon | 90 | 0.5 | 8.6 |
| 176 | malathion | 87 | 2.5 | 6.2 |
| 177 | mandipropamide | 83 | 2.5 | 2.8 |
| 178 | MCPA | 86 | 2.5 | 4.1 |
| 179 | MCPB | 83 | 125 | 1.6 |
| 180 | mecarbam | 87 | 0.5 | 9.0 |
| 181 | mecoprop | 82 | 5 | 6.6 |
| 182 | mefenpyr-diethyl | 84 | 2.5 | 8.7 |
| 183 | mepanipyrim | 81 | 0.5 | 2.9 |
| 184 | mepronil | 80 | 1.25 | 6.2 |
| 185 | metaflumizone | 70 | 50 | 11.0 |
| 186 | metalaxyl | 86 | 5 | 1.0 |
| 187 | metamitron | 85 | 5 | 4.1 |
| 188 | metazachlor | 90 | 0.5 | 9.7 |
| 189 | metconazole | 83 | 2.5 | 6.4 |
| 190 | methacriphos | 102 | 50 | 4.8 |
| 191 | methamidophos | 72 | 12.5 | 7.7 |
| 192 | methidathion | 90 | 2.5 | 1.2 |
| 193 | methiocarb | 88 | 5 | 5.9 |
| 194 | methiocarb-sulfone | 89 | 0.5 | 2.7 |
| 195 | methiocarb sulfoxide | 86 | 0.5 | 7.2 |
| 196 | metholcarb | 92 | 12.5 | 5.5 |
| 197 | methomyl | 89 | 25 | 4.6 |
| 198 | methoxyfenozide | 89 | 50 | 7.5 |
| 199 | metobromuron | 88 | 2.5 | 9.3 |
| 200 | metolachlor | 92 | 0.5 | 5.4 |
| 201 | metosulam | 91 | 1.25 | 9.7 |
| 202 | metoxuron | 86 | 0.5 | 8.0 |
| 203 | metribuzin | 89 | 0.5 | 5.7 |
| 204 | metsulfuron-methyl | 94 | 2.5 | 3.0 |
| 205 | mevinphos | 89 | 1.25 | 3.9 |
| 206 | monocrotophos | 92 | 0.5 | 7.3 |
| 207 | monolinuron | 87 | 1.25 | 2.0 |
| 208 | monuron | 91 | 1.25 | 7.7 |
| 209 | myclobutanil | 90 | 0.5 | 9.4 |
| 210 | naled | 81 | 50 | 7.8 |
| 211 | napropamide | 90 | 0.5 | 5.6 |
| 212 | neburon | 87 | 1.25 | 2.4 |
| 213 | nicosulfuron | 94 | 0.5 | 7.5 |
| 214 | norflurazone | 90 | 0.5 | 3.2 |
| 215 | omethoate | 86 | 1.25 | 4.3 |
| 216 | oxadixyl | 90 | 2.5 | 9.3 |
| 217 | oxamyl | 90 | 2.5 | 8.0 |
| 218 | oxydemeton-methyl | 92 | 1.25 | 5.5 |
| 219 | oxyfluorfen | 91 | 250 | 6.7 |
| 220 | paclobutrazol | 83 | 2.5 | 3.8 |
| 221 | penconazole | 86 | 1.25 | 2.8 |
| 222 | pencycuron | 89 | 2.5 | 7.0 |
| 223 | pendimethalin | 88 | 50 | 4.0 |
| 224 | permethrin | 82 | 125 | 3.2 |
| 225 | phenmedipham | 90 | 0.5 | 7.6 |
| 226 | phenothrin | 83 | 5 | 0.8 |
| 227 | phenthoate | 90 | 1.25 | 7.1 |
| 228 | phorate | 79 | 250 | 6.8 |
| 229 | phorate-sulfone | 94 | 1.25 | 5.4 |
| 230 | phorate-sulfoxide | 95 | 125 | 6.7 |
| 231 | phosalone | 102 | 2.5 | 11.5 |
| 232 | phosmet | 91 | 1.25 | 1.2 |
| 233 | phosphamidon | 96 | 0.5 | 4.2 |
| 234 | phoxim | 75 | 1.25 | 2.5 |
| 235 | picloram | 85 | 125 | 6.9 |
| 236 | picolinafen | 93 | 5 | 5.8 |
| 237 | picoxystrobin | 90 | 1.25 | 3.4 |
| 238 | pinoxaden | 93 | 0.5 | 9.1 |
| 239 | piperonyl butoxide | 90 | 10 | 2.1 |
| 240 | pirimicarb | 90 | 5 | 6.4 |
| 241 | pirimiphos-ethyl | 88 | 0.5 | 8.3 |
| 242 | pirimiphos-methyl | 88 | 1 | 3.7 |
| 243 | prochloraz | 82 | 5 | 6.4 |
| 244 | profenofos | 87 | 5 | 2.1 |
| 245 | prometon | 88 | 0.5 | 1.3 |
| 246 | prometryn | 83 | 0.5 | 8.5 |
| 247 | propachlor | 88 | 0.5 | 6.2 |
| 248 | propamocarb | 76 | 5 | 4.7 |
| 249 | propaquizafop | 83 | 2.5 | 9.3 |
| 250 | propargite | 86 | 1.25 | 5.7 |
| 251 | propazine | 84 | 0.5 | 3.3 |
| 252 | propham | 85 | 250 | 7.1 |
| 253 | propiconazole | 88 | 12.5 | 6.5 |
| 254 | propoxur | 88 | 12.5 | 4.3 |
| 255 | propoxycarbazone | 89 | 12.5 | 5.0 |
| 256 | propyzamide | 81 | 12.5 | 8.7 |
| 257 | proquinazid | 70 | 0.5 | 7.6 |
| 258 | prosulfocarb | 80 | 0.5 | 5.0 |
| 259 | prothioconazole-desthio | 81 | 1.25 | 4.4 |
| 260 | prothiofos | 84 | 500 | 4.6 |
| 261 | pyraclostrobin | 85 | 1.25 | 8.5 |
| 262 | pyrazophos | 86 | 0.5 | 6.0 |
| 263 | pyrethrin I | 82 | 25 | 3.0 |
| 264 | pyrethrin II | 88 | 125 | 7.7 |
| 265 | pyridaben | 76 | 1.25 | 7.4 |
| 266 | pyridate | 89 | 0.5 | 9.5 |
| 267 | pyrifenox | 82 | 2.5 | 9.7 |
| 268 | pyrimethanil | 87 | 1.25 | 7.3 |
| 269 | pyriproxyfen | 83 | 0.5 | 7.4 |
| 270 | quinalphos | 88 | 2.5 | 2.7 |
| 271 | quinclorac | 88 | 5 | 2.9 |
| 272 | quinmerac | 88 | 0.5 | 7.5 |
| 273 | quinoclamine | 87 | 5 | 4.1 |
| 274 | quinoxyfen | 86 | 12.5 | 7.3 |
| 275 | quizalofop | 88 | 25 | 6.8 |
| 276 | quizalofop-p-ethyl | 88 | 2.5 | 2.3 |
| 277 | resmethrin | 75 | 2.5 | 6.5 |
| 278 | rimsulfuron | 78 | 2.5 | 8.6 |
| 279 | rotenone | 87 | 2.5 | 10.7 |
| 280 | simazine | 90 | 0.5 | 3.9 |
| 281 | simetryn | 84 | 0.5 | 8.7 |
| 282 | spinosyn A | 78 | 2.5 | 7.8 |
| 283 | spinosyn D | 72 | 12.5 | 9.1 |
| 284 | spirodiclofen | 89 | 2.5 | 8.1 |
| 285 | spiromesifen | 91 | 5 | 5.7 |
| 286 | spiroxamine | 73 | 0.5 | 7.9 |
| 287 | sulfosulfuron | 86 | 2.5 | 5.2 |
| 288 | sulfotep | 93 | 1.25 | 5.1 |
| 289 | tau-fluvalinate | 83 | 500 | 5.3 |
| 290 | tebuconazole | 101 | 0.5 | 5.2 |
| 291 | tebufenozide | 90 | 12.5 | 6.8 |
| 292 | tebufenpyrad | 83 | 2.5 | 7.8 |
| 293 | teflubenzuron | 88 | 250 | 2.2 |
| 294 | tepraloxydim | 90 | 2.5 | 7.1 |
| 295 | terbufos | 88 | 250 | 8.0 |
| 296 | terbufos-sulfone | 87 | 1.25 | 5.3 |
| 297 | terbufos-sulfoxide | 89 | 0.5 | 3.9 |
| 298 | terbuthylazine | 88 | 0.5 | 9.5 |
| 299 | terbutryn | 86 | 0.5 | 8.5 |
| 300 | tetraconazole | 88 | 2.5 | 4.8 |
| 301 | tetramethrin | 88 | 2.5 | 7.6 |
| 302 | thiabendazole | 75 | 0.5 | 1.3 |
| 303 | thiacloprid | 86 | 0.5 | 9.1 |
| 304 | thiamethoxam | 88 | 5 | 8.2 |
| 305 | thifensulfuron-methyl | 92 | 1.25 | 7.1 |
| 306 | thiodicarb | 82 | 0.5 | 10.0 |
| 307 | thiometon | 90 | 125 | 6.1 |
| 308 | thiophanate-methyl | 78 | 1.25 | 7.6 |
| 309 | tolclofos-methyl | 93 | 250 | 7.6 |
| 310 | tolylfluanid | 89 | 5 | 8.2 |
| 311 | triadimefon | 90 | 1.25 | 6.5 |
| 312 | triadimenol | 94 | 5 | 1.8 |
| 313 | triasulfuron | 96 | 2.5 | 4.9 |
| 314 | triazophos | 91 | 0.5 | 5.3 |
| 315 | trichlorfon | 92 | 1.25 | 8.4 |
| 316 | tricyclazole | 85 | 0.5 | 3.8 |
| 317 | trifloxystrobin | 97 | 1.25 | 9.5 |
| 318 | triflumuron | 94 | 50 | 10.3 |
| 319 | triforine | 76 | 500 | 7.8 |
| 320 | trinexapac ethyl | 90 | 5 | 3.5 |
| 321 | triticonazole | 84 | 2.5 | 6.3 |
| 322 | vamidothion | 81 | 0.5 | 5.2 |
| 323 | zoxamide | 89 | 2.5 | 4.1 |

**Table S3. Concentrations of silymarin flavonoids / flavonolignans determined in the milk thistle preparations**

| **Sample No.** | **Sampling year** | **Sample code*** | **Taxifolin** | **Silychristin** | **Silydianin** | **Silybin A** | **Silybin B** | **Isosilybin**  **A** | **Isosilybin**  **B** | **2,3-Dehydrosilybin (A+B)** | **Sum of flavonoid / flavonolignans** |
| --- | --- | --- | --- | --- | --- | --- | --- | --- | --- | --- | --- |
| **1** | 2016 | **USA 1-I** | 5.3 | 35.9 | 14.0 | 59.1 | 73.2 | 17.7 | 8.0 | 2.4 | **216** |
| **2** | 2017 | **USA 1-II** | 7.0 | 40.6 | 12.6 | 70.7 | 84.6 | 19.1 | 6.9 | 1.2 | **243** |
| **3** | 2016 | **USA 2** | 0.4 | 4.1 | 0.6 | 6.6 | 6.0 | 1.8 | 0.8 | 0.5 | **21** |
| **4** | 2016 | **USA 3-I** | 5.1 | 31.8 | 9.6 | 46.6 | 58.4 | 13.9 | 4.9 | 1.0 | **171** |
| **5** | 2017 | **USA 3-II** | 7.9 | 38.2 | 6.2 | 27.8 | 38.6 | 15.5 | 4.8 | 1.9 | **141** |
| **6** | 2016 | **USA 4-I** | 4.8 | 31.5 | 8.8 | 18.8 | 27.2 | 13.4 | 5.3 | 1.2 | **111** |
| **7** | 2017 | **USA 4-II** | 9.0 | 38.1 | 13.3 | 25.1 | 37.2 | 16.2 | 6.0 | 1.3 | **146** |
| **8** | 2017 | **USA 4-III** | 8.6 | 38.0 | 12.3 | 42.2 | 54.0 | 15.5 | 5.7 | 1.2 | **177** |
| **9** | 2017 | **USA 4-IV** | 0.6 | 4.9 | 1.7 | 6.6 | 6.8 | 2.3 | 0.9 | 0.4 | **24** |
| **10** | 2016 | **USA 5-I** | 11.0 | 66.8 | 34.8 | 105.5 | 130.2 | 30.9 | 11.7 | 1.6 | **393** |
| **11** | 2017 | **USA 5-II** | 12.9 | 58.9 | 27.4 | 92.4 | 111.3 | 27.7 | 10.3 | 1.5 | **342** |
| **12** | 2017 | **USA 5-III** | 9.9 | 60.3 | 36.2 | 93.2 | 114.1 | 30.1 | 7.1 | 1.9 | **353** |
| **13** | 2016 | **USA 6-I** | 6.1 | 44.2 | 21.6 | 41.3 | 60.0 | 20.1 | 8.9 | 1.4 | **204** |
| **14** | 2017 | **USA 6-II** | 10.7 | 42.8 | 12.1 | 69.0 | 81.8 | 18.8 | 6.3 | 0.8 | **242** |
| **15** | 2017 | **USA 6-III** | 11.6 | 46.6 | 13.1 | 74.0 | 87.8 | 20.0 | 7.3 | 0.9 | **261** |
| **16** | 2017 | **USA 6-IV** | 12.2 | 51.6 | 15.9 | 31.4 | 52.9 | 20.4 | 7.2 | 1.6 | **193** |
| **17** | 2017 | **USA 7-I** | 8.8 | 34.5 | 9.9 | 51.9 | 62.9 | 13.6 | 5.1 | 0.8 | **188** |
| **18** | 2017 | **USA 7-II** | 7.7 | 30.5 | 12.1 | 41.5 | 50.9 | 12.8 | 5.0 | 0.9 | **161** |
| **19** | 2017 | **USA 8** | 9.3 | 39.6 | 15.3 | 64.2 | 76.4 | 17.5 | 6.4 | 1.1 | **230** |
| **20** | 2016 | **CZ 1** | 6.7 | 49.7 | 13.4 | 89.4 | 110.7 | 24.0 | 8.0 | 0.9 | **303** |
| **21** | 2016 | **CZ 2** | 4.3 | 39.8 | 13.3 | 17.3 | 29.0 | 18.0 | 6.6 | 1.3 | **130** |
| **22** | 2016 | **CZ 3** | 2.0 | 12.2 | 4.7 | 21.6 | 21.5 | 5.5 | 1.9 | 0.5 | **70** |
| **23** | 2016 | **CZ 4** | 0.5 | 3.7 | 2.0 | 4.4 | 5.2 | 1.4 | 0.5 | 0.0 | **18** |
| **24** | 2016 | **CZ 5** | 0.1 | 1.0 | 0.6 | 1.1 | 1.9 | 0.5 | 0.2 | 0.0 | **5** |
| **25** | 2016 | **CZ 6** | 1.2 | 9.2 | 4.3 | 13.4 | 13.8 | 4.3 | 1.7 | 0.4 | **49** |
| **26** | 2016 | **CZ 7** | 3.0 | 17.5 | 8.2 | 28.1 | 32.0 | 8.2 | 3.0 | 0.9 | **101** |

Values represent mg of individual flavonoids / flavonolignans per g of the particular milk thistle preparation

*Sample code: country of origin, brand- batch No

**Table S4. European Union maximum limits for mycotoxins in certain food commodities**

| **Deoxynivalenol**^1^ | **Concentration** (µg/kg) |
| --- | --- |
| Unprocessed cereals other than durum wheat, oats and maize | 1,250 |
| Unprocessed durum wheat and oats | 1,750 |
| Unprocessed maize | 1,750 |
| Cereals intended for direct human consumption, cereal flour, bran and germ as end product marketed for direct human consumption, with the exception of foodstuffs listed above | 750 |
| Pasta (dry) | 750 |
| Bread (including small bakery wares), pastries, biscuits, cereal snacks and breakfast cereals | 500 |
| Processed cereal-based foods and baby foods for infants and young children | 200 |
| **Zearalenone**^1^ |  |
| Unprocessed cereals other than maize | 100 |
| Unprocessed maize with the exception of unprocessed maize intended to be processed by wet milling | 350 |
| Cereals intended for direct human consumption, cereal flour, bran and germ as end product marketed for direct human consumption, with the exception of foodstuffs listed above | 75 |
| Refined maize oil | 400 |
| Bread (including small bakery wares), pastries, biscuits, cereal snacks and breakfast cereals, excluding maize snacks and maize-based breakfast cereals | 50 |
| Maize intended for direct human consumption, maize-based snacks and maize-based breakfast cereals | 100 |
| Processed cereal-based foods (excluding processed maize-based foods) and baby foods for infants and young children | 20 |
| Processed maize-based foods for infants and young children | 20 |
| **sum of HT2 and T2 toxins (HT2 + T2)^2^** |  |
| unprocessed barley (including malting barley) and maize / oats/ wheat, rye and other cereals | 200 / 1,000 / 100 |
| oats / maize / other cereals for direct human consumption | 200 / 100 / 50 |
| oat bran and flaked oats | 200 |
| cereal bran except oat bran, oat milling products other than oat bran and flaked oats, and maize milling products | 100 |
| other cereal milling products | 50 |
| breakfast cereals including formed cereal flakes | 75 |
| bread (including small bakery wares), pastries, biscuits, cereal snacks, pasta | 25 |
| cereal-based foods for infants and young children | 15 |

^1^ COMMISSION REGULATION (EC) No 1881/2006 of 19 December 2006 setting maximum levels for certain contaminants in foodstuffs

^2^ COMMISSION RECOMMENDATION of 27 March 2013 on the presence of T-2 and HT-2 toxin in cereals and cereal products

**Table S5. Quantification of microbial species identified in the milk thistle preparations**

| **Sample No.** | **Sampling year** | **Sample code *** | **Microbial contamination**  [CFU (colony forming unit) / g] |
| --- | --- | --- | --- |
| **1** | 2016 | **USA 1-I** | *Cladosporium* *tenuissimum* (9), *Aspergillus* *tubingensis* (11) |
| **2** | 2017 | **USA 1-II** | *Paecilomyces* *variotii* (84), *Bacillus* *pumilus* (8), *Monascus* *purpureus* (36), *Aspergillus* *montevidensis* (8) |
| **3** | 2016 | **USA 2** | *Chaetomium* *globosum* (12), *Bacillus* *subtilis* (6) |
| **4** | 2016 | **USA 3-I** | *Cladosporium* *tenuissimum* (6) |
| **5** | 2017 | **USA 3-II** | *Paracoccus* *yeei* (7), *Massilia* *timonae* (50), *Cryptococcus* *saitoi* (8), *Cryptococcus* *liquefaciens* (10) |
| **6** | 2016 | **USA 4-I** | *Aspergillus* *sydowii* (32), *Bacillus* *pumilus* (15), *Aspergillus* *montevidensis* (10), *Paenibacillus* *illinoisensis* (7), *Aspergillus* *microperforatus* (11), *Scopulariopsis* *cinerea* (10), *Aspergillus* *versicolor* (11) |
| **7** | 2017 | **USA 4-II** | *Streptomyces* *griseus* (12), *Cladosporium* *tenuissimum* (12), *Pantoea* *agglomerans* (10), *Cryptococcus* *diffluens* (12) |
| **8** | 2017 | **USA 4-III** | *Staphylococcus* *capitis* (5), *Cryptococcus* *liquefaciens* (6) |
| **9** | 2017 | **USA 4-IV** | *Bacillus* *subtilis* (5), *Monascus* *purpureus* (5) |
| **10** | 2016 | **USA 5-I** | *Paecilomyces* *variotii* (7), *Aspergillus* *pseudoglaucus* (9), *Aspergillus* *ruber* (9) |
| **11** | 2017 | **USA 5-II** | *Aspergillus* *fumigatus* (9) |
| **12** | 2017 | **USA 5-III** | *Aspergillus* *pseudoglaucus* (14) |
| **13** | 2016 | **USA 6-I** | *Paecilomyces* *variotii* (87), *Bacillus* *megaterium* (38), *Bacillus* *endophyticus* (77), *Cryptococcus* *diffluens* (13), *Bacillus* *simplex* (13), *Aspergillus* *pseudoglaucus* (10), *Bacillus* *pumilus* (58), *Gracibacillus* *diposauri* (74), *Bacillus* *subtilis* (179), *Paenibacillus* *jamilae* (29), *Bacillus* *licheniformis* (15), *Paenibacillus* *amylolyticus* (13), *Bacillus* *sonorensis* (13), *Aspergillus* *sydowii* (19), *Paenibacillus* *glucanolyticus* (13) |
| **14** | 2017 | **USA 6-II** | *Cladosporium* *tenuissimum* (11) |
| **15** | 2017 | **USA 6-III** | *Aspergillus* *microperforatus* (56), *Aspergillus* *montevidensis* (28) |
| **16** | 2017 | **USA 6-IV** | *Bacillus* *pumilus* (37), *Aspergillus* *tubingensis* (54), *Penicillium* *palitans* (13) |
| **17** | 2017 | **USA 7-I** | *Aspergillus* *microperforatus* (7), *Aspergillus* *terreus* (7), *Bacillus* *pumilus* (18), *Cladosporium* *tenuissimum* (7) |
| **18** | 2017 | **USA 7-II** | no contamination |
| **19** | 2017 | **USA 8** | *Bacillus* *subtilis* (6), *Escherichia* *vulneris* (11), *Aspergillus* *montevidensis* (6), *Pseudomonas* *oryzihabitans* (6) |

**Figure S1. Structures and summary formulas of silymarin flavonoid/flavonolignans**


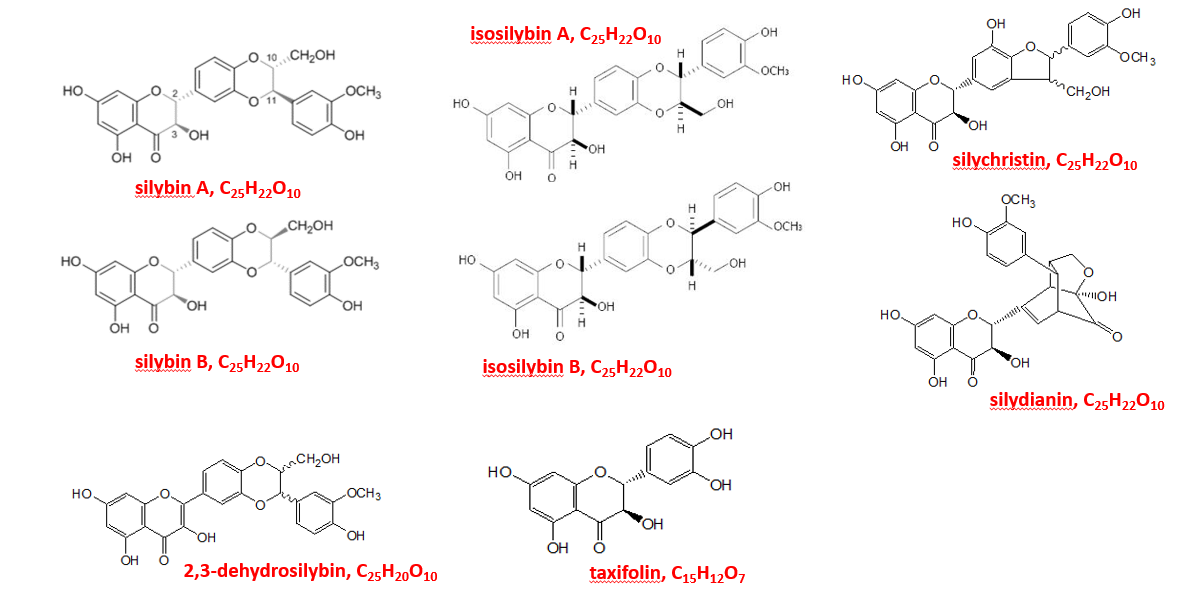


**Figure S2. U-HPLC-HRMS chromatogram of silymarin flavonoid/flavonolignans present in the internal reference sample of milk thistle-based dietary supplement.**

The reversed phase AccucoreTM aQ analytical column (150mm x 2.1mm; i.d. 2.6 µm; Thermo Scientific, San Jose, CA, USA) maintained at 40°C was used for the gradient elution, where the mobile phases consisted of H_2_O : MeOH (98:2, v/v) (A) and MeOH : H2O (98:2, v/v) (B), both containing 10 mM ammonium acetate and 0.2 % formic acid. The gradient was as follows: 30 % of B (initial), linear increase to 41 % of B (in 0.5 min) held for 8.5 min, another linear increase to 100 % of B (in 0.5 min) held for 3 min and final column equilibration for 3 min under the initial conditions. The mobile phase flow rate was 0.3mLmin-1 and the injection volume 2 µL.

The mass spectrometer operated in the in the full MS mode at following parameters: electrospray ionization in negative mode (ESI-), sheath and auxiliary gas flow rates 45 and 10 arbitrary units, respectively; spray voltage 3.5 kV; heater temperature 300°C; capillary temperature 320 °C, and S-lens RF level 55. Following parameters were used in full MS mode: mass resolving power 70,000 FWHM (defined for m/z 200; 3 Hz), scan range 80–1200 m/z, automatic gain control (AGC) target 3e6, maximum inject time (IT) 100 ms, and in the dd-MS/MS mode: intensity threshold 1e4, resolution 17,500 FWHM (defined for m/z 200; 12 Hz), scan range 50 – fragmented mass m/z (m/z +25), AGC target 5e4, maximum IT 50 ms, normalized collision energy (NCE) 35 % with _20 % step, isolation window 1 m/z and dynamic exclusion of fragmented analyte for 5 s. For the method development and data evaluation, XcaliburTM and Q-Exactive Tune software (Thermo Scientific, San Jose, CA, USA) were used. The following exact masses were considered for detection: 303.0510 ([M-H]^-^ ions of taxifolin), 479.0984 ([M-H]^-^ ions of 2,3-dehydrosilybin) and 481.1140 ([M-H]^-^ ions of the other isomeric flavonolignans).


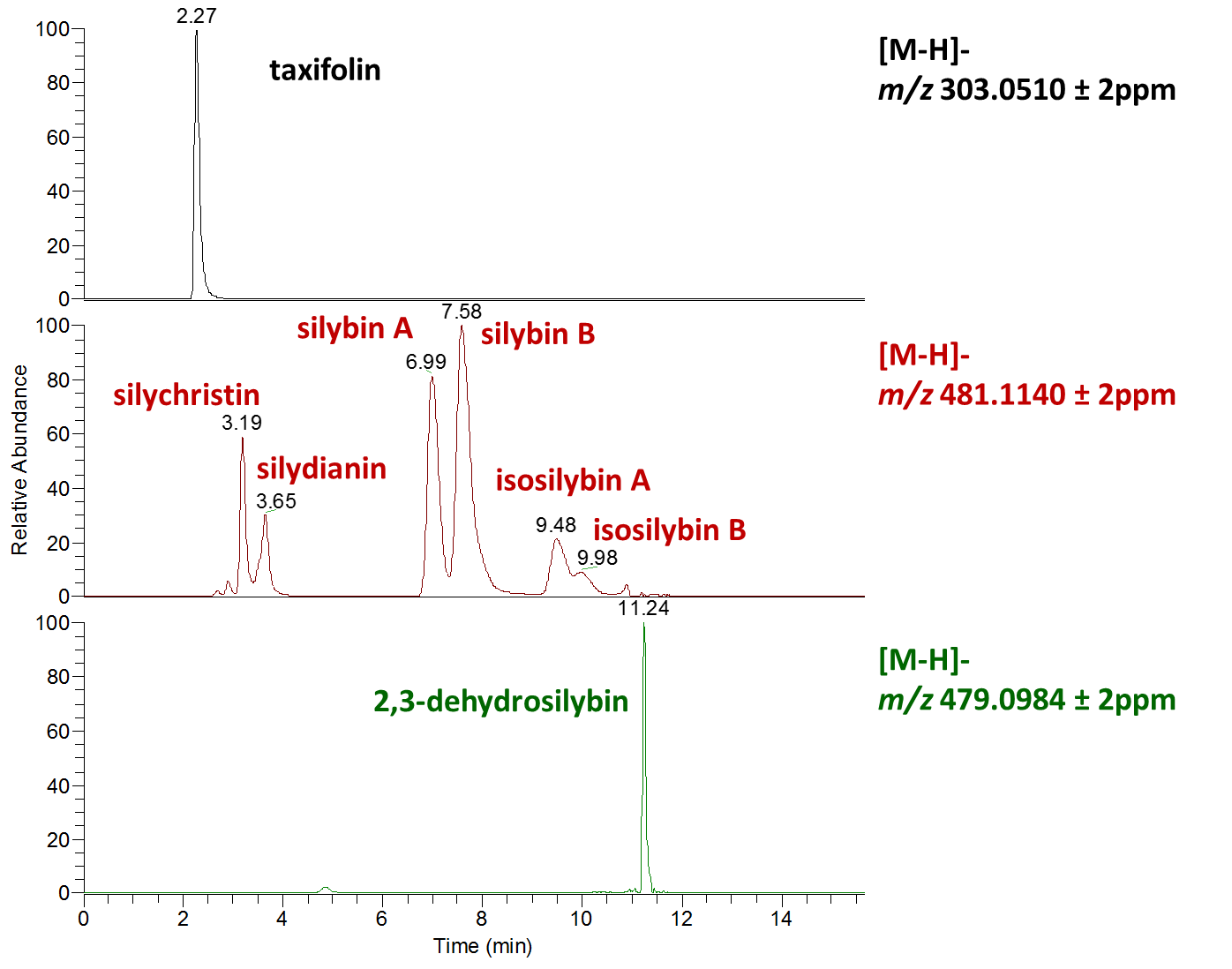

Supplement: Supplementary file 1 — Dataset 1 [file 41598_2019_47250_MOESM1_ESM.docx]
